# Synthesis of Ladder and Flexible Polysulfonimides Directly From Anionic Monomers: Investigating Structure–Property Convergence in Succinonitrile–Polymer Electrolyte Blends

**DOI:** 10.1002/anie.8257644

**Published:** 2026-09-26

**Authors:** Abraham Herzog‐Arbeitman, Pablo Leon, Benedikt S. Schreib, KyuJung Jun, Jurgis Ruza, Vanesa Munoz Perales, Yang Shao‐Horn, Rafael Gomez‐Bombarelli, Jeremiah A. Johnson

**Affiliations:** ^1^ Department of Chemistry Massachusetts Institute of Technology Cambridge Massachusetts USA; ^2^ Department of Materials Science and Engineering Massachusetts Institute of Technology Cambridge Massachusetts USA

**Keywords:** electrolyte, ionic conductivity, microporous polymer, polymer of intrinsic microporosity, porosity, single‐ion conductor, succinonitrile

## Abstract

Insights into how permanent microporosity influences ion transport in polymer electrolytes could facilitate the design of next‐generation single‐ion conducting (SIC) materials. Intrinsically microporous polymers and conventional polymers are typically regarded as fundamentally distinct electrolyte platforms; direct comparisons between compositionally similar examples are lacking. Here, we introduce a strategy for the synthesis of fluorinated aryl sulfonimide‐based polymers of intrinsic microporosity (FAS‐PIMs) together with flexible polysulfonimide analogs of similar composition that lack permanent microporosity. Although these polymers possess fundamentally different dry‐state architectures, incorporation of a common pore‐filling transport medium, succinonitrile (SN), drives the two systems toward remarkably similar nanostructures with similar bulk conductivities (>10^−5^–10^−4^ S/cm at 40°C–80°C), thermomechanical properties (*G*ʹ within 1–10 MPa over the temperature range of 25°C–120°C), and reprocessability. Molecular dynamics simulations and experiments suggest these unusual combinations of properties arise through different conduction mechanisms within the materials, and that sulfonimide anions are particularly advantageous for conduction in microporous matrices relative to strongly binding anions. Overall, this work introduces the first sulfonimide‐based PIM, establishes a comparison between permanently microporous and transiently porous single‐ion conducting polymers, and demonstrates that these two traditionally distinct electrolyte architectures can converge toward similar, useful bulk properties after incorporation of a pore‐filling transport medium.

## Graphical abstract

Direct synthesis of sulfonimide‐based polymers of intrinsic microporosity enables comparison with compositionally similar flexible analogs. Incorporation of a common pore‐filling transport medium drives these fundamentally different polymer architectures toward similar bulk conductivity and mechanical properties while revealing distinct microscopic ion‐transport mechanisms.

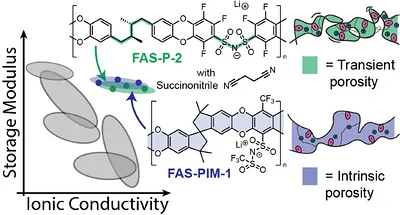

**Abbreviations**
NMR: Nuclear Magnetic Resonance

## 1 Introduction

Porous polymeric materials, including polymers of intrinsic microporosity (PIMs), covalent organic frameworks (COFs) [1], and metal–organic frameworks (MOFs) [2], have shown great promise for size‐selective filtration of gasses and other small molecules thanks to their tunable, interconnected micropore structures [3, 4]. When filled with ionic solutions, these materials can alter ion solvation and diffusion via competitive interactions with the matrix, nanoconfinement effects, and size‐sieving [5, 6, 7]. Of the various classes of these materials, high molecular‐weight PIMs can offer facile (re)processability when not chemically cross‐linked, robust mechanical properties, improved transference numbers of liquid electrolytes relative to bulk electrolyte [8, 9], and ion selective transport [8, 10, 11]. Nevertheless, PIMs incorporating ion‐solvating [10] and charged groups have only recently been explicitly designed for electrochemical applications. For example, PIMs with anionic pendants have displayed near‐unity transference numbers in aqueous ion‐exchange membranes [12], improving the lifetime and performance of aqueous zinc cells relative to cells based on Nafion and Celgard membranes. Yet, prior studies involving solvent‐filled porous material electrolytes generally assumed that permanent microporosity is advantageous without comparing these materials to compositionally similar variants that lack permanent porosity. Conventional polymers are well known to swell upon solvent uptake, creating transient free‐volume elements capable of supporting ion transport. This raises a broader question: after incorporation of a common pore‐filling transport medium, how do permanently microporous polymers and solvent‐swollen conventional polymers differ in structure and properties? Addressing this question requires direct comparisons between compositionally related porous and non‐permanently porous polyanions, which have been largely absent due to synthetic limitations.

The reported synthetic routes to charged PIMs (including cationic PIMs [13]) so far involve post‐polymerization modification of neutral PIMs (e.g., via direct sulfonation [14] or nitrile hydrolysis/aminolysis [15, 16, 17] for anionic PIM‐1 analogues), leading to additional synthetic steps to introduce anionic pendants, undesired degradation [18], and limited anion diversity. Moreover, the anions employed thus far, which include carboxylates [5], amidoximates [19], and sulfonates [14], are substantially more Lewis basic than the weakly coordinating anions commonly used in battery electrolytes and therefore bind cations like Li^+^ and Na^+^ relatively strongly outside of highly polar solvents like water. We envisioned that PIMs bearing sulfonimide‐based anionic pendants [20, 21, 22] similar to conventional battery electrolyte components could provide weaker ion pairing to promote ion transport in non‐aqueous electrolytes.

For example, we wondered whether weakly coordinating anionic PIMs, when combined with a pore‐filling medium that facilitates ion hopping, could simultaneously achieve high ionic conductivity and robust thermomechanical properties. Mechanical stiffness and conductivity are often opposing features in polymer‐based electrolytes [23], with useful conductivities (>10^−5^ S/cm) often only observed above the polymer *T*_g_ or melting temperature, that is, when the electrolyte is very soft or even liquid‐like. For example, blends of PEO and LiTFSI, which feature amongst the highest conductivities of all reported polymer electrolytes [24], only achieve conductivities >10^−3^ S/cm well above their melting temperatures where they have low moduli (tensile storage modulus <1 MPa above ≈60°C) [25, 26] and viscous, liquid‐like character [27]. This tradeoff may be disrupted by supramolecular self‐assembly [28], engineered coplanar hydrogen bonding [29], mechanically interlocked networks [30], or by filling materials such as ionic MOFs [31], and COFs [1] with coordinating liquids or polymers. While this manuscript was under evaluation, independent studies on polyanions with ionic liquid fillers showed simultaneous improvement of mechanical modulus alongside conductivity when rigid polymer backbones were present, although these materials are not single‐ion conductors [32].

If sulfonimide‐based PIMs could be prepared with high molecular weights to promote entanglement and then combined with (nonionic) pore‐filling additives that promote ion hopping, the resulting composites could exhibit an unusual combination of high single‐ion conductivity, mechanical stiffness, thermal stability, and reprocessability. Furthermore, by replacing the rigid ladder backbone with more flexible repeat units containing rotatable sp^3^–sp^3^ or sp^3^–sp^2^ bonds, insight into the consequences of permanent versus transient porosity in polymer electrolytes could be obtained.

Here, we describe the synthesis of fluorinated aryl sulfonimide‐based PIMs (FAS‐PIMs) together with compositionally similar, flexible polysulfonimide analogs through direct nucleophilic aromatic substitution (S_N_Ar) polymerization of fluorinated aryl sulfonimide tagged (FAST) anion monomers. Optimized polymerization conditions provide high molecular weight polymers of both classes capable of forming free‐standing membranes. By comparing these polymers before and after incorporation of a common pore‐filling transport medium, succinonitrile (SN), we investigate whether permanent microporosity remains a defining structural feature of the resulting composite electrolyte or whether controlled swelling drives convergence toward similar bulk structures and properties despite potentially distinct microscopic transport mechanisms. When 5 equivalents of SN are incorporated into both polymers, the resulting composites, regardless of whether they derive from permanently microporous or flexible polymer precursors, display Li^+^ conductivities σ_Li_ (>10^−5^ S/cm at 30°C) among the highest reported for SIC polymers while simultaneously exhibiting solid‐like mechanical behavior (*G*′ of ∼1–10 MPa), thermal stability, and reprocessability. Overall, this work introduces a direct synthetic route to sulfonimide‐based PIMs from ionic monomers and provides a framework for comparing permanently microporous and transiently porous polymer electrolytes after incorporation of a common pore‐filling transport medium.

## 2 Results

### 2.1 Synthesis of FAS‐PIMs and Related Polysulfonimides From FAST Salt Monomers

We first set out to synthesize PIMs with dissociative sulfonimide pendants. Sulfonimides are widely used as salt anions in batteries (e.g., LiTFSI and NaTFSI) and have lower pK_a_ values (increasing dissociation constant) in organic solvents than their sulfonate, carboxylate, or amidoximate counterparts [33]. Indeed, direct comparisons between sulfonates and sulfonimides in blends of SIC polymers and polyethylene oxide have shown significant gains in conductivity for the latter [34]. While COF and MOF electrolytes been prepared from various anionic linkers, including sulfonimides [35], such anions have not been employed in readily processable PIMs.

To achieve this goal, we proposed that FAST anions [36, 37], which were originally developed as a platform to access a diverse range of Li^+^, Na^+^, and related salts via S_N_Ar reactions, could potentially be used as novel building blocks for PIM synthesis, see Figure 1A. In particular, “FAST‐B,” which contains 4 reactive aromatic fluorides as well as trifluoromethyl and sulfonimide substituents, could potentially serve as an electrophile for S_N_Ar polymerization with tetramethyl‐tetrahydroxyspirobisindane (TTSBI), a commercially available, contorted bis‐catechol used as a building block for the canonical “PIM‐1.” While replacement of the aryl fluorides with catechol‐like substituents raises the Lewis basicity of the FAST anion relative to FAST‐B (increasing the calculated dissociation energies by ≈30 kJ/mol in vacuum, see Figure S1), the strongly electron withdrawing CF_3_ group and resonance stabilized sulfonimide is nevertheless expected to lead to greater cation mobility compared to reported PIM‐1‐derived polyanions that have carboxylate, sulfonate, and related ionic pendants.

**FIGURE 1:**
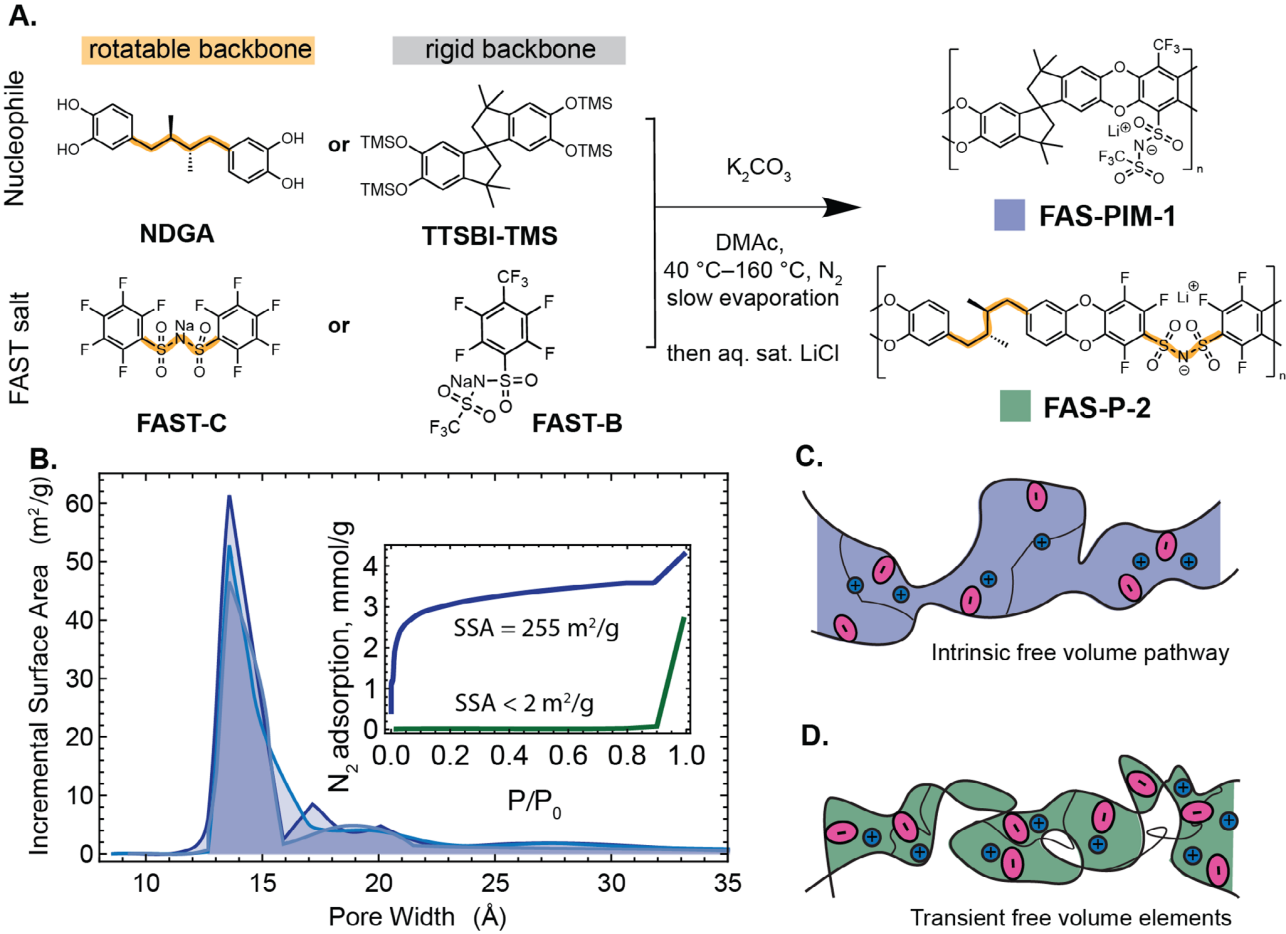
Synthesis of FAS polymers. (A) Synthetic scheme for FAS‐PIM‐1 and FAS‐P‐2 discussed in this work. (B) Pore distribution of FAS‐PIM‐1 (three measurements shown). Inset: N_2_ adsorption isotherms at 77 K for FAS‐PIM‐1 and FAS‐P‐2. (C) Schematic for free volume and micropore structure in FAS‐PIM‐1. (D) Schematic for free volume and micropore structure in FAS‐P‐2.

We set out to develop conditions for the polymerization of **FAST‐B** (Na^+^ salt) with **TTSBI** to provide an anionic polysulfonimide—**FAS‐PIM‐1**. Initially, we explored conditions analogous to commonly employed PIM‐1 syntheses, wherein the comonomers are treated with K_2_CO_3_ either at 60°C for up to several days [38] or at higher temperatures such as 160°C for 1 h [39]; however, while the expected reactions do proceed, the oxidative instability of TTSBI resulted in broad and poorly reproducible product molecular weights and molecular weight distributions. Changing other conditions like concentration, solvent composition (dimethyl formamide vs. anhydrous dimethyl acetamide), temperature (60°C–160°C), and atmosphere (positive or neutral nitrogen pressure) were similarly unsuccessful and/or not reproducible. Eventually, we found that exhaustive silylation of TTSBI alongside slow solvent evaporation under positive nitrogen pressure during polymerization yielded high molecular weight Na^+^ and K^+^‐based polymers (*M_n_* ≈ 1.40 × 10^5^ − 1.65 × 10^5^; *Đ* ≈ 1.4–1.8 as determined by size‐exclusion chromatography, Figure S2). Here, silylation suppresses TTSBI oxidation and quenches fluoride generated in situ during S_N_Ar polymerization [40], improving reproducibility and enabling complete monomer conversion (as determined by ^19^F NMR; Figure S3). The resulting crude polymer was subjected to cation exchange conditions using aqueous LiCl and dialysis, providing the Li^+^‐based polymer (Figure 1A) in 77% overall isolated yield after drying at 140°C under vacuum (see Supporting Information for full details); no Na^+^, K^+^, or Cl^–^ was observed by ^23^Na NMR and x‐ray photoelectron spectroscopy after dialysis (Figure S4). Thermogravimetric analysis (TGA) suggested that the polymer is thermally stable to at least >200°C (Figure S5A). Differential scanning calorimetry (DSC) displayed no thermal transitions in the range of −40°C–140°C (Figure S5b), consistent with expectations for rigid ladder polymers. Samples of lower molecular weight were isolated for later comparisons (*vide infra*) by quenching the polymerization before complete monomer conversion.

The two‐dimensional small‐angle x‐ray scattering (SAXS) profile obtained from a film of this polymer displayed a broad peak at *q* = 0.35 Å^−1^, corresponding to a periodic distance *d* = 1.79 nm, which is larger than the monomer repeat unit dimensions (≈1.5 nm); we attribute this peak to the presence of micropores within the bulk material (Figure S6). This feature shifts to higher *q* values for low molecular weight (*M_n_* ≈ 2 × 10^3^) samples, corresponding to smaller characteristic length scales as would be expected for free volume elements partially filled by polymer end‐groups [41]. We note that other microporous polymers of comparable molecular weights, such as carboxylated PIM‐1 (*q* = 0.35 Å^−1^), display similar SAXS profiles [5]. Finally, N_2_ adsorption isotherms at 77 K indicate that this material exhibits significant intrinsic microporosity, with an accessible Brunauer–Emmet–Teller (BET) surface area of 255 m^2^/g (Figure 1B). Based on these results, we refer to this lithiated polymer as “**FAS‐PIM‐1Li**,” as it is the first sulfonimide‐based PIM, to our knowledge.

Seeking to compare the impacts of backbone flexibility and subsequent porosity on SIC properties, similar copolymerization and cation exchange conditions were applied to the monomers “**FAST‐C**,” which possesses two pentafluorophenyl substituents in place of the trifluoromethyl groups of TFSI, and nordihydroguaiaretic acid (**NDGA**), providing a Li^+^‐based polymer “**FAS‐P‐2Li**” in 52% yield. SEC (Figure S2) showed that while **FAS‐P‐2Li** has a similar weigh‐average molar mass (*M*_w_) compared to **FAS‐PIM‐1Li**, its molar mass dispersity (*Ð*) is substantially larger, which may reflect the formation of branched and/or low molecular weight cyclic species facilitated by the greater flexibility of the **FAS‐P‐2Li** backbone. Nevertheless, TGA and DSC of **FAS‐P‐2Li** showed similar thermal behaviors compared to **FAS‐PIM‐1Li** (Figure S5). Moreover, while a broad feature at *q* = 0.26 Å^−1^ (*d* = 2.38 nm, Figure S6) was observed in the SAXS profile of **FAS‐P‐2Li**, which might result from the length of a repeat unit (2.25 nm), ion aggregates, and/or free‐volume elements, N_2_ adsorption isotherms at 77 K suggest that this polymer has no accessible permanent microporosity (specific surface area < 2 m^2^/g). While **FAS‐P‐2Li** has a similar atomic composition, mass per repeat unit, and ion exchange capacity compared to **FAS‐PIM‐1Li**, the absence of permanent microporosity can be attributed to the presence of several rotatable single bonds per repeat unit in its backbone [42]. Finally, we note that another polysulfonimide, **FAS‐P‐3Li**, was prepared from **NDGA** and **FAST‐B**; however, we were unable to obtain high molecular weights for this polymer under the conditions employed, and therefore we focused on **FAS‐PIM‐1Li** and **FAS‐P‐2Li** for the remainder of this investigation.

### 2.2 Polysulfonimide–Succinonitrile Composites: Rationale, Fabrication, and Thermomechanical Properties

Anionic polymers often display negligible (<10^−8^ S/cm) bulk cation conductivities due in part to the significant distances between their anionic binding sites [43, 44]. Similarly, **FAS‐PIM‐1Li** displayed negligible conductivity in the bulk state (Figure S7). In the context of previously reported anionic PIMs [5, 17], water has been used as a solvent and pore filler to facilitate cation (K^+^ and Li^+^) mobility, leading to promising aqueous electrolytes with conductivities above 30 mS/cm; however, organic fillers used to promote ion transport have not been widely investigated. Considering the possible organic additives that could be used in this regard, we identified succinonitrile (SN) as a promising starting point. SN is a plastic crystal that, when blended with lithium salts, displays superionic conductivity due to a proposed “paddlewheel” mechanism wherein rapid C─C bond rotation within SN molecules facilitates ion transport [45, 46, 47]. While SN itself is a solid with a melting temperature >50°C, blending it with salts and polymers [48, 49, 50, 51] that disrupt its crystal structure can lead to high conductivities [52, 53, 54] but typically generates plasticized materials with lower melting points and thus liquid‐like behaviors at moderate temperatures [52]. Here, we hypothesized that the entangled, microporous architecture of **FAS‐PIM‐1Li** could serve as a scaffold to distribute SN throughout its free‐volume network while preserving the mechanical integrity of the polymer framework (Figure 1C). We further wondered whether a compositionally similar, non‐permanently porous polymer might evolve toward a comparable composite structure after controlled swelling with the same pore‐filling transport medium (Figure 1D). Having synthetic access to both **FAS‐PIM‐1Li** and **FAS‐P‐2Li** therefore provides an opportunity to investigate whether these two fundamentally different dry‐state polymer architectures remain distinct after SN incorporation or instead converge toward similar bulk composite structures.

**FAS‐PIM‐1Li** and **FAS‐P‐2Li** were dissolved in acetone and combined with 5 molar equivalents of SN per repeat unit (≈36.5 wt.%). This ratio was selected to provide sufficient SN to solvate Li^+^ (4 molar equivalents), plus a slight excess, without compromising mechanical properties. The acetone was then removed under high vacuum without measurable SN loss as confirmed by ^1^H NMR spectroscopy (Figure S8), providing composite materials **C1** and **C2**, respectively, which could be cast into freestanding films. SAXS analysis of **C1** revealed a broad peak between *q* = 0.5–0.07 Å^−1^, *d* = 1.2–9.0 nm), consistent with formation of a hierarchy of micro‐ and mesopores, and no detectable crystalline peaks in the WAXS region (Figure S9). **C2** displayed similar features (Figure S9), with the addition of a peak at *q* = 1.4 Å^−1^, *d* = 4.5 Å in the WAXS region that may indicate the presence of some crystalline SN in this sample. Molecular dynamics (MD) simulations of **C1** and **C2** show similar pore sizes and size distributions (Figure S10) along with similar densities (Figure S11). Finally, despite having ≈36.5 wt% SN, no SN melting transitions were observed by DSC for either **C1** or **C2** (Figure S5B) over the range of −40°C–140°C, consistent with disruption of the plastic crystalline structure of bulk SN and distribution of the SN within the swollen polymer frameworks. While **C1** did not display any discernible glass transitions, **C2** did show a weak glass transition at 20°C, which we attribute to plasticization of **FAS‐P‐2Li** by SN.

Small‐angle oscillatory shear rheology was used to characterize the thermomechanical properties of **C1** and **C2** (Figures 2A, S12, and S13). Neat SN exhibits a roughly 6 order‐of‐magnitude decrease in shear storage modulus (*G*ʹ) upon passing through its melting point, consistent with a solid‐to‐liquid‐like transition. By contrast, **C1** and **C2** displayed high *G*ʹ between 1–10 MPa, with *G*ʹ always at least 5‐fold greater than the corresponding loss moduli, *G*ʹʹ, for **C2** and 10‐fold for **C1**. These materials thus display viscoelastic solid‐like behavior at all temperatures tested (from room temperature up to 120°C) and less viscous flow than solid‐like supramolecular materials [55]. Frequency sweeps collected at 25°C and 120°C (Figure S13) further support these results, showing similar moduli and no crossover of *G*ʹ and *G*ʹʹ, that is, no transition to viscous fluid‐like behavior, at all tested frequencies. For context, the storage moduli of **C1** and **C2** are similar to common plastics and cross‐linked elastomers like silica‐filled silicones [56], soft polyurethanes [57], plasticized PVC [58], and vulcanized rubber [59]. We hypothesize that even though **C2** is above its *T*_g_, its rheological response at experimental timescales is dominated by entanglements and SN‐salt interactions, creating properties similar to **C1**.

**FIGURE 2:**
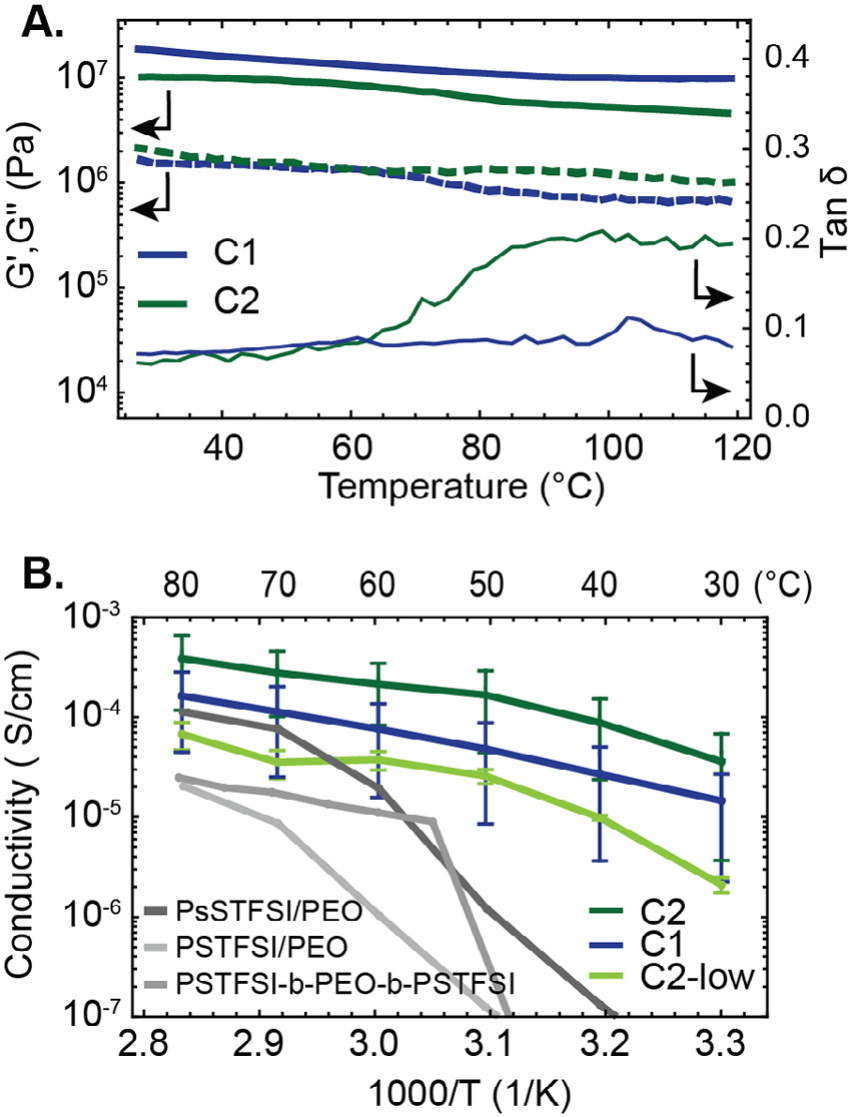
Conductivity and mechanical properties of **C1**, **C2** and other polsulfonimide/SN composites for comparison. (A) Representative storage modulus (*G*ʹ, thick solid lines), loss modulus (Gʹʹ, thick dashed lines) and loss tangent (tan δ, thin solid lines) temperature sweeps for **C1** and **C2** composites at 10 Hz. Replicates and average curves can be found in the Supporting Information. (B) Conductivity of **C1** and **C2** with a low molecular weight variant, “**C2‐low”** and Li single‐ion conducting polymer material benchmarks for comparison. PsSTFSI/PEO [70], (dark gray); PSTFSI/PEO [70] (light gray); PSTFSI‐b‐PEO‐b‐PSTFSI [62] (medium gray) all include sulfonimide anions and PEO to facilitate conduction either as a blend or block component. Full data can be found in Figure S19.

Both materials retained ≈50% of their room‐temperature *G*ʹ at 120°C (40% ± 16% for **C2**, 65% ± 12% for **C1**), suggesting they could withstand local heating in batteries without substantial softening (Figure S12). Finally, *G*ʹ for both **C1** and **C2** composites remained stable through two heating and cooling cycles (from room temperature to 120°C, Figure S13), indicating that SN did not phase separate or evaporate and that the microstructures of **C1** and **C2** are thermally stable within this regime. These data, together with the SAXS, WAXS, and DSC results, suggest that **C1** and **C2** comprise entangled polymer frameworks containing polymer‐associated SN distributed throughout swollen free‐volume domains. Although the dry‐state architectures differ substantially, the resulting composite nanostructures share important structural similarities; however, **C1**, unlike **C2**, does not appear to be plasticized upon SN addition, and it retains a higher storage modulus and lower loss tangent, reflecting the locked conformation of the ladderlike backbone.

Finally, flammability and (re)proccessability—critical considerations for next‐generation electrolyte materials—were preliminarily investigated for **C1**. When exposed to an open flame (Figure S14), **C1** charred slowly but did not ignite under the conditions tested. Moreover, **C1** could be remolded into new form factors under mild conditions (65°C, 2 tons of pressure, 5 min), or its components could be reclaimed in moderate yield through direct sublimation of SN under reduced pressure at 65°C and recovery of pure, high molecular weight **FAS‐PIM‐1Li** (following precipitation, ≈ 40% yield) (Figure S15).

### 2.3 **C1** and **C2** Display High Conductivities and Single‐Ion Conducting Character

In most uncross‐linked polymer materials, dopants can rapidly diminish mechanical properties. For example, addition of SN to PEO/LiTFSI materials is known to increase conductivity but also soften the materials substantially [50]. Figure S16 shows that at the same polymer content as **C1**, PEO/LiTFSI/SN materials have ≈400x lower storage modulus at 30°C and lose integrity completely around 40°C. By contrast, **C1** and **C2** retain their elastic solid behaviors and high moduli while exhibiting conductivities >10^−5^ S/cm at 30°C and 10^−4^ S/cm at 80°C (Figure 2B). The single‐ion conducting character of **C1** was examined using the Bruce‐Vincent method (see Supporting Information), yielding an apparent $\;t_{Li^{+}}$ of 0.85 ± 0.1 (see Figure S17). The Bruce–Vincent method assumes highly dilute and non‐interacting charge carrier concentrations [60, 61], an assumption that is violated in these complex composite materials; thus, this value should be taken as qualitative. Notwithstanding, the observed value is qualitatively consistent with single‐ion conducting character expected from highly entangled polyanions and with transference numbers extracted from molecular dynamics simulations (vide infra).

These Li^+^ conductivities are among the highest reported for SIC polymer materials with shear storage moduli of ∼10 MPa, surpassing well‐known benchmarks like poly(styrene‐sulfonimides, PSTFSI) [62] and poly[(4‐styrenesulfonyl)(trifluoromethyl(S‐trifluooromethylsulfonylimino)sulfonyl)imide], PSsTFSI) [34]. The transference number of **C2**, as inferred from molecular dynamics simulations, was observed to be 0.9. To our knowledge, the only other materials with comparable reported transference numbers, conductivities, and moduli of similar magnitudes are cross‐linked [63, 64], precluding their facile (re)processing, or are unentangled supramolecular materials vulnerable to flowing over time [65].

Figure S18 compares the property profiles of **C1** and **C2** with exemplary Li single‐ion conducting materials reported in the literature. **C1** and **C2** retain favorable reprocessability and temperature stability, alongside moduli comparable to glassy polymer nanoparticle composites [23], which are ≈100 times less conductive. When carbonate solvents are added to different anionic nanoparticles, similar conductivities can be achieved but *G*ʹ remains >10x lower and temperature stability suffers [66]. Conversely, **C1** and **C2** show conductivity comparable to soft ionic oligomer pastes (i.e., liquids), while retaining stable performance over a temperature window >100°C and >3 orders of magnitude higher moduli [67]. While other poly(sulfonimide) electrolytes have shown similar conductivities to **C1** and **C2** alongside engineered reprocessability using carbonate and sulfolane plasticizers, their reported transference numbers are below 0.7, with >10x lower modulus and higher loss tangents [68] or no discussion of mechanical properties [69].

### 2.4 Mechanistic Insights Into the Performance of **C2**

Despite originating from fundamentally different dry‐state polymer architectures, **C1** and **C2** exhibit remarkably similar conductivity and thermomechanical properties after incorporation of SN. We hypothesized that entanglement of the polymer components of **C1** and **C2** is critical to forming a continuous load‐bearing framework that confines SN. To test this hypothesis, an analog of **C2** was prepared, referred to as “**C2‐low**,” using low molecular weight **FAS‐P‐2Li** (M*_n_* = 17 kDa; *Đ* = 2.0). While the room temperature conductivity and moduli of **C2‐low** were similar to **C1** and **C2** above, dramatic melting transitions were observed around 40°C distinguished by ≈6‐orders‐of‐magnitude decreases in *G*ʹ and crossovers of *G*ʹ and *G*ʹʹ to liquid‐like behavior, consistent with the melting of SN domains and the lack of an entangled polymer matrix (Figure S20). Thus, high molecular weight is crucial to maintaining the dimensional stability of **C2** materials at high temperatures. Notably, however, the conductivities of this liquid **C2** analog do not exceed those of **C1** *or* **C2** within the temperature range investigated, despite the latter being solids with effectively immobile anions. This observation is consistent with our picture of **C1** and **C2** forming percolated networks of disordered SN molecules, the latter coordinating to Li^+^ and facilitating its diffusion. It also provides further evidence that polymer entanglement, rather than, for example, ionic interactions between polymers chains, is the primary source of mechanical stability in **C1** and **C2**, as the disruption of ionic aggregates at higher temperatures should significantly increase conductivity and reduce stiffness, which is not observed. Finally, the slightly decreased conductivities observed for **C2‐low** can be attributed to suppressed dissociation of Li^+^ from the sulfonimides in these polymers, which is supported by FTIR spectra showing a diminished blue‐shifted nitrile stretching peak at ≈2290 cm^−1^ of SN coordinated to Li^+^ [71] for **C2‐low** compared to **C1** and **C2** (Figure S21).

### 2.5 Diffusion Mechanisms of Li^+^ Polysulfonimide/SN Composites

To investigate mechanisms of ion conduction within **C1** and **C2**, the local solvation structure and dynamics of Li^+^ were interrogated through MD simulations. The simulations provide a molecular explanation for how **C1** and **C2** converge toward similar bulk properties while retaining distinct local coordination environments. Tracking of Li^+^ diffusion over 50 ns at 80°C yielded Li^+^ conductivity values (Nernst‐Einstein and cluster Nernst–Einstein) of (2.73 ± 0.504) × 10^−4^ S/cm for **C1** and (2.03 ± 0.427) × 10^−4^ S/cm for **C2** (Figure 3A,B) that agreed well with the experimental values at 80°C. The overall diffusivities of Li^+^, SN, and the FAS backbones (defined by tracking backbone ether oxygens) for both **C1** and **C2** were calculated from the simulated mean squared displacements in the diffusive regime (Figure 3A). Li^+^ and SN were observed to diffuse 6 and 13 times faster than the **FAS‐PIM‐1** backbone, respectively, giving a high simulated transference number ≈0.95 qualitatively consistent with experiment. Li^+^ diffused slightly faster within **C2** simulations and anion motion was again negligible.

**FIGURE 3:**
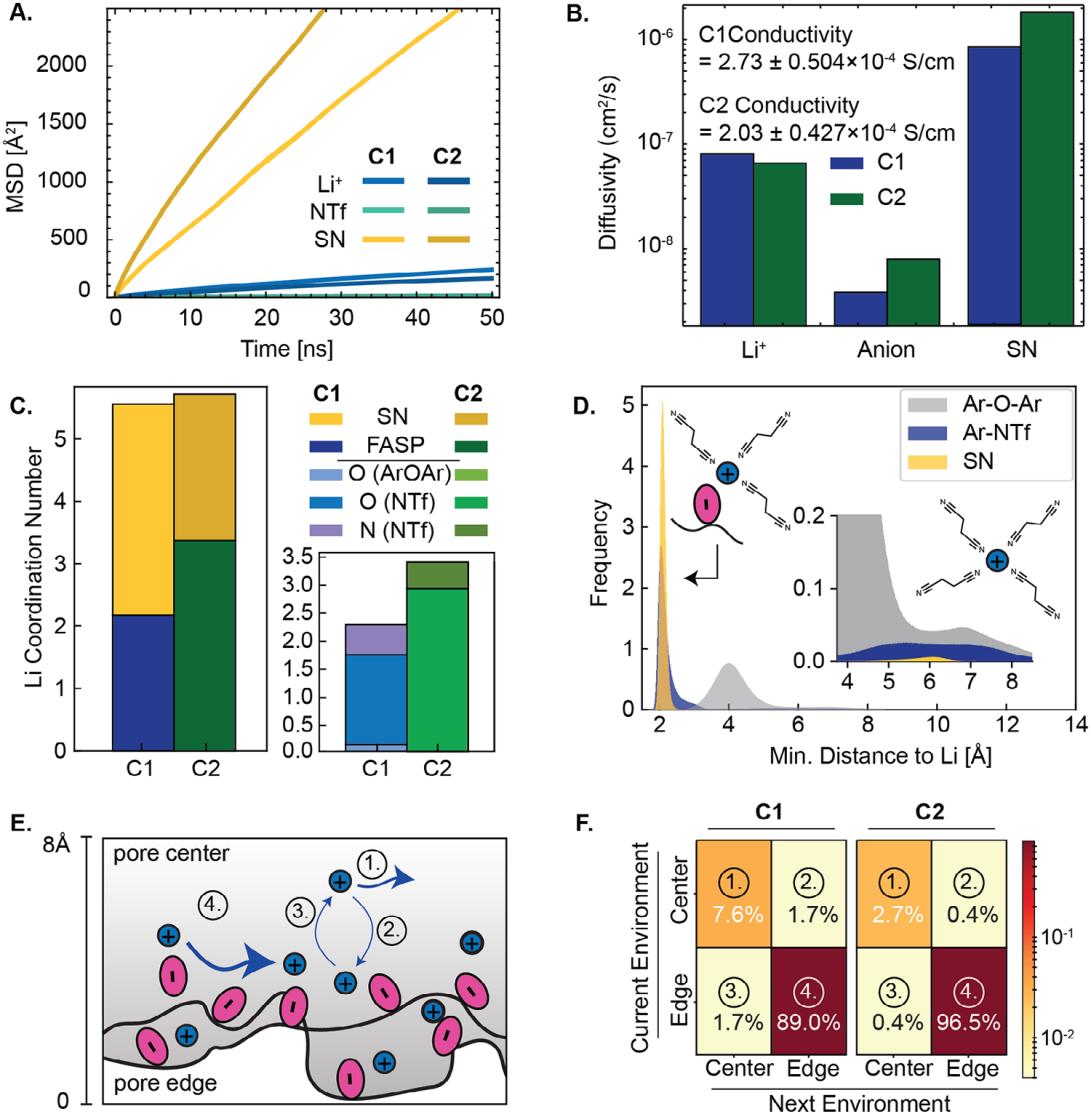
Li^+^ coordination environment and dynamics. (A) Mean squared displacement of SN (yellow), Li^+^ (blue), and polymer (green) within the first 50 ns of a 100 ns simulation for **C1** and **C2** (see Methods for details). (B) Extracted diffusivities of **C1** and **C2** components. Extracted diffusivities for FAS‐PIM‐1 and FAS‐P‐2 “anions” are effectively negligible. (C) Breakdown of Li^+^ coordination sphere in **C1** and **C2**. FASP refers to either FAS‐PIM‐1 or FAS‐P‐2. The rightmost plot shows contributions of different heteroatoms within the polymer backbones to SN coordination. (D) Distance distribution between Li^+^ and possible coordination partners. Inset: subpopulation of Li^+^ far from polymer‐bound sulfonimides (i.e., in the pore center). (E) Schematic of simulated pore structure showing Li^+^ at model‐derived pore edge and in the model‐derived pore center, and the diffusion in and between these environments. Numbers label diffusion within and between these environments (F) Quantifications of Li^+^ diffusion between different environments via processes illustrated in (E). Quadrant colors and labeled percentages indicate the fraction of Li^+^ undergoing a given transition in or between environments. Exact values depend on the timescale at which these processes are compared, see Methods.

Examination of simulated Li^+^ solvation environments revealed competitive coordination of Li^+^ by the pore‐confined SN and sulfonimides from the FAS backbones, with more Li^+^ coordinated by SN relative to the backbone in **C1** vs. **C2** (Figure 3C). In **C1**, the majority of simulated Li^+^ ions are coordinated by a single sulfonimide and 2, 3, or 4 SN (average ≈3.3) molecules completing the solvation shell. 9% of Li^+^ are solely coordinated by SN, and no Li^+^ were observed to be solely coordinated by **FAS‐PIM‐1**, underscoring the greater solvation strength of SN relative to the dissociative sulfonimide anions. In **C2**, sulfonimides make up a greater proportion of all coordination interactions, with only 3% of Li^+^ completely solvated by SN, suggesting that the rigid **FAS‐PIM‐1** backbone discourages ionic aggregation relative to the flexible **FAS‐P‐2**, which can more readily chelate cations. Examination of the simulated Li^+^ radial distribution functions (RDFs, see Figure S22) and the distributions of Li‐backbone, Li‐anion, and Li‐SN distances (shown in Figure 3D for **C1** and Figure S23 for **C2**) indicates that while Li^+^ is generally in close proximity to SN, the Li^+^‐sulfonimide distance distribution (defined as the Li‐N distance) showed weak bimodality in SN composites, with a minor peak at 5–7 Å (half the average pore diameter), corresponding to a Li^+^ subpopulation fully coordinated by SN in the **C1** pore center. Tracking the movement of Li ions within and between these environments, shown schematically in Figure 3E, indicates that diffusion from the pore edge to the pore center or vice versa is relatively rare (Figure 3E,F), that is, Li^+^ atoms that hop from the pore edge into the pore center typically spend a significant amount of time there before returning to sulfonimide coordination at the edge (Figure 3F).

Simulated Li^+^ diffusion within the model‐derived pore center and at the model‐derived pore edge is observed to occur by qualitatively different mechanisms and differs between **C1** and **C2** in accordance with their different thermal properties. A typical sulfonimide‐bound Li^+^ time course in **C2** is shown in Figure 4A: Li^+^ can hop to other anion coordination sites when anions come within ≈5 Å, but this process does not necessarily coincide with significant Li^+^ displacement. After hopping, the displacement versus time graph for Li^+^ matches that of its coordinating anion, indicating diffusion coupled to polymer segmental motion as is common for plasticized polymer electrolytes. By contrast, Figures 4B and S24 show qualitatively different behavior for an analogous edge‐bound Li^+^ in the ladderlike **C1**: here, Li^+^ moves only in discrete hops between anions, enabled by sulfonimide pendant rotation, and does not undergo significant diffusion without exchanging anionic sites. Hopping in the simulations is facilitated by the adjacent SN coordination; in simulations without SN, Li^+^ is bound tightly to sulfonimide oxygens (Figure S22) and displays negligible conductivity consistent with experiment (Figure S7). This pore‐edge sulfonimide “handoff” mechanism is likely only possible in minimally swollen networks, where charged chains remain relatively close to each other. These findings underscore thermal and rheological characterizations of **C1** and **C2** (vide supra) that find differing effects of SN in matrices of differing rigidity. In **C2**, SN acts as a plasticizer, decreasing T_g_ into the measurable range, slightly increasing tan δ above 60°C, and increasing the rate of **FAS‐P‐2** segmental motion in simulation. In **C1**, segmental motion is not observed to increase, no *T*_g_ is observed, and tan δ remains low up to 120°C. Thus, unlike in **C2**, SN does not act as a plasticizer in **C1**, instead increasing conductivity through means other than backbone segmental motion (i.e., as an ion transporter independent of the polymer). Despite these differences, **C1** and **C2** converge on relatively similar thermomechanical and conductive profiles within the studied temperature range.

**FIGURE 4:**
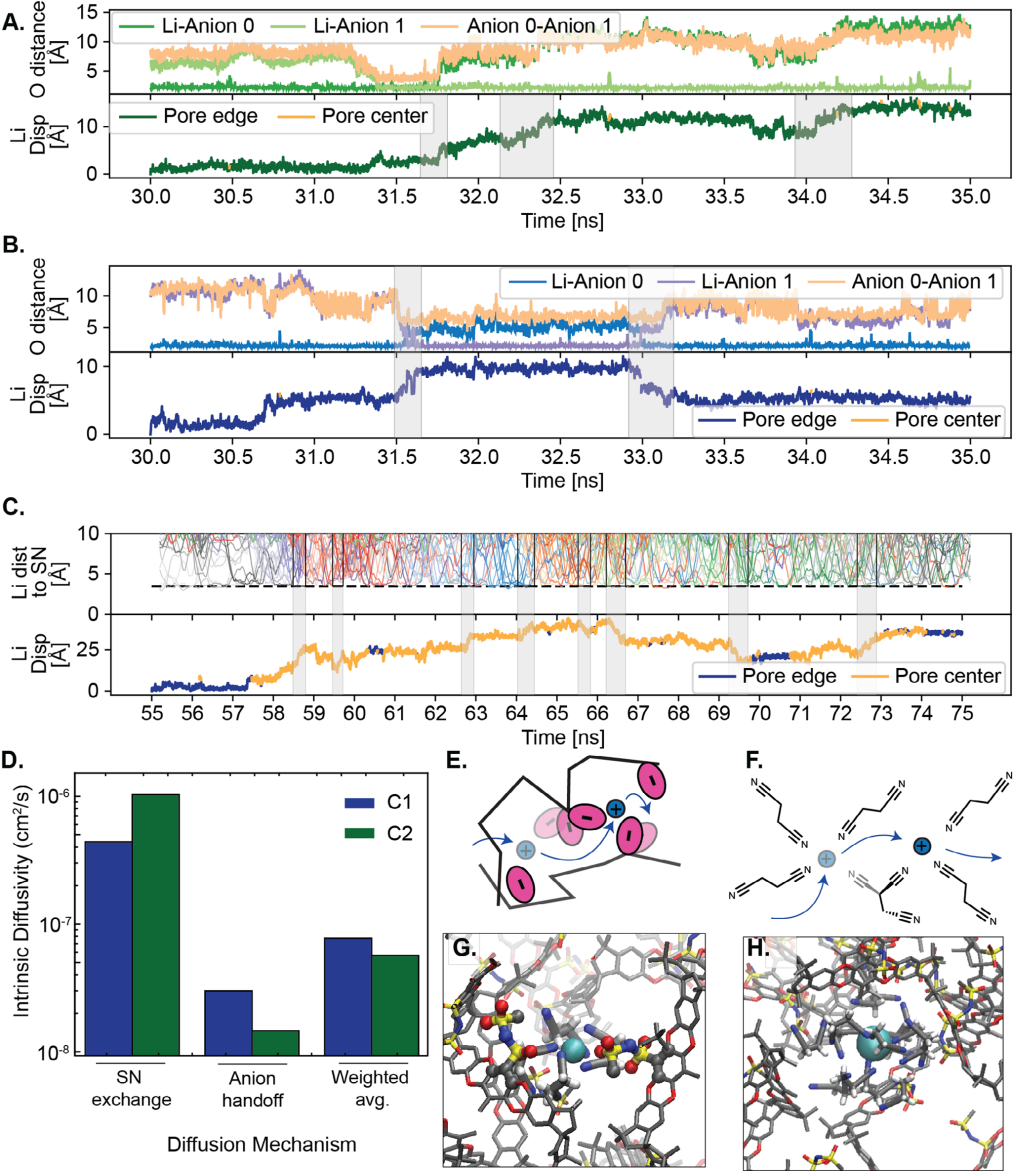
Li^+^ diffusion mechanisms in different environments. Timecourses are shown for Li^+^ traveling along the pore edge in **C2** (A) via segmental motion, along the pore edge via sulfonimide handoff in **C1** (B), and predominantly in the pore center of **C1** (C). Li^+^‐ligand distances over time (top in A–C) track coordination partners to show changes in the coordination environment over time. Each coordinating species is a different color. Gray highlights show periods of rapid Li^+^ diffusion, which do not coincide with drops in the anion‐anion distance in (A) but do in (B). Periods of rapid Li^+^ diffusion correlate with significant ligand exchange in (C). (D) Intrinsic Li^+^ diffusivities (normalized by frequency of event) of different conduction mechanisms in **C1** and **C2**. (E–H) Schematics and simulation snapshots of pore‐edge sulfonimide handoffs and pore‐center SN exchange in **C1**. For further details, see Supporting Information.

In the model‐derived pore centers of **C1** and **C2**, Li^+^ diffuses primarily via SN–SN exchange/hopping, as demonstrated in Figures 4C, S25, and S26. In **C1** simulations, Li^+^ is coordinated consistently by 4–6 SN molecules at a time and interacts with over 100 SN molecules over its time in the pore center (≈20 ns). Jumps of several Å are evident within the Li displacement time course, taking place within ≈0.5 ns and coinciding with the exchange of several SN at a time. These events are consistent with Li^+^ hopping between binding pockets within the confined SN phase. When the coordination sphere is relatively static (regions of consistent color and low slope in the SN‐Li^+^ distance curves), Li^+^ diffusion is significantly slower and Li^+^ typically moves less than 1 Å before changing its coordination sphere, indicating vehicular diffusion does not contribute substantially to displacement relative to hopping. Discrete hops can also be observed within the **C2** time course (Figure S26), but more vehicular diffusion can be seen as well, consistent with more mobile, liquid‐like SN phase within the matrix.

Normalizing the diffusion contributions of each mechanism by its occurrence frequency yields intrinsic diffusivities (Figure 4D), showing that SN‐based diffusion is more rapid than sulfonimide‐mediated mechanisms in our simulations. Hopping relies on facile dissociation of cations from polymer‐bound anions, underscoring the utility of highly dissociative sulfonimide anions (relative to more common carboxylates, etc.), especially in rigid microporous matrices which enable cations to take advantage of free‐volume pathways within the matrix. Schematic depictions of these mechanisms and representative snapshots of each in **C1** are shown in Figure 4E–H. Despite the rapid diffusion of pore‐center localized Li^+^ ions, the dominant contribution to ionic conductivity derives from Li^+^ hopping along the pore edge as shown in Figure S27 due to the much greater concentration of charge carriers bound to sulfonimide. Overall, these results suggest that further increases in ion conductivity could be realized in similar systems in the future by decreasing the distance between anionic sites on the polymer backbone and further lowering the anion solvation strength, both of which should lower the energy barrier to sulfonimide hopping. Pore‐filling additives besides SN may enhance this mechanism; however, further swelling the matrix similarly to conventional gels is likely to isolate anionic sites and discourage this conduction pathway.

Finally, we note that the simulations described above employ a non‐polarizable force field with partial charges scaled to 0.9, following common practice for solid polymer electrolytes (Figure S28) [72, 73, 74, 75]. This empirical treatment reproduces the experimentally observed structural features and conductivity trends, but it is expected to influence ion pairing, coordination environments, and the relative frequencies of the model‐derived transport mechanisms. Consequently, the mechanistic pathways presented here should be interpreted as model‐derived hypotheses consistent with the experimental observations rather than uniquely established transport mechanisms.

## 3 Conclusion

The successful synthesis of **FAS‐PIM‐1** establishes the first, to our knowledge, sulfonimide‐based polymer of intrinsic microporosity prepared directly from ionic monomers, providing a new route to permanently microporous single‐ion conducting polymers with greater ionic dissociativity than previous carboxylate, sulfonate, and amidoximate polymers. Comparison with a compositionally similar, flexible polysulfonimide demonstrates that these fundamentally different dry‐state polymer architectures converge toward remarkably similar bulk conductivity, thermomechanical properties, and reprocessability after incorporation of a common pore‐filling transport medium, including a combination of conductivity and mechanical properties that has been difficult to achieve [76].

At the same time, convergence of the bulk properties does not imply convergence of the underlying transport mechanisms. Spectroscopic measurements and molecular dynamics simulations suggest that permanent microporosity alters the local Li^+^ coordination environment and the relative contributions of model‐derived transport pathways, with the rigid **FAS‐PIM** framework promoting a larger population of Li^+^ ions coordinated predominantly by succinonitrile and greater contributions from pore‐center diffusion, whereas the flexible polysulfonimide relies more strongly on plasticization and polymer segmental motion. We cannot exclude the potential contributions of slight differences in the sulfonimide structure and substitution between flexible and microporous polymers in creating this mechanistic divergence. Regardless, the ability of these different conduction mechanisms to create similar conductivities is surprising. These observations suggest that permanent microporosity remains an important design variable for tuning ion transport mechanisms even when bulk properties converge, and that anion identity is crucial in mediating these properties.

We highlight the explicit role of the polar dopant as a rich opportunity for further work. Highly polar yet semisolid nitrile plastic crystals like SN and higher dinitriles [77] have demonstrated promising transport properties, including in the present composites; however, their known instability toward lithium metal [78] remains a significant limitation for practical battery applications. Although passivating interfacial layers such as LiF or others may mitigate this issue under some conditions, such as those we employ in this work (Figure S29), more electrochemically stable pore‐filling additives will likely be required for practical Li‐metal batteries.

More broadly, this work suggests that solvent‐filled permanently microporous polymers and solvent‐swollen conventional polymers should not necessarily be viewed as fundamentally distinct classes of electrolyte materials. Instead, incorporation of a common pore‐filling transport medium can drive convergence toward similar bulk composite structures while preserving distinct microscopic transport behavior. We anticipate that understanding this convergence will provide a useful framework for designing future single‐ion conducting polymer electrolytes in which polymer structure, ion coordination, and pore‐filling additives are engineered together to optimize performance.

## 4 Experimental Section

### 4.1 Materials

Common solvents (dichloromethane, dimethyl acetamide, hexane, ethyl acetate, acetone, and toluene) were purchased from Sigma‐Aldrich. 4‐Trifluoromethyl, 2,3,5,6‐tetrafluorobenzene thiol was purchased from Ambeed Bedpharm Inc., and used as received. Trifluoromethanesulfonamide was purchased from Fischer Sci. and used as received. Succinonitrile was purchased from Fischer Scientific and sublimed twice before use. All other reagents were purchased from Sigma‐Aldrich and were used without further purification unless otherwise noted. All deuterated solvents were purchased from Cambridge Isotope Laboratories. FAST‐C was synthesized using a literature procedure [20]. 4‐Trifluoromethyl, 2,3,5,6‐tetrafluorobenzene sulfonyl bromide was synthesized using a literature procedure [79]. Tetra(trimethylsilyl) 5,5',6,6'‐tetrahydroxy‐3,3,3',3'‐tetramethyl‐1,1'‐spirobisindane (TTSBI‐TMS) was synthesized according to a literature procedure [40] and distilled prior to use.

### 4.2 Methods

#### 4.2.1 Column Chromatography

Flash silica column chromatography was performed using Biotage Isolera One with Accelerated Chromatographic IsolationTM flash chromatography system using KP‐Sil SNAP cartridges at recommended flow rates.

#### 4.2.2 Solution‐State Nuclear Magnetic Resonance (NMR) Spectroscopy

^1^H and ^13^C spectra were collected at rt (25°C) and recorded on either a two‐channel Bruker Avance‐III HD Nanobay 400 MHz spectrometer, three‐channel Bruker Avance Neo 500 MHz spectrometer, or four‐channel Bruker Avance Neo 600 MHz spectrometer. Data were analyzed with MestReNova Version: 14.1.0‐24037. Chemical shifts are expressed in parts per million (ppm), and splitting patterns are designated as s (singlet), d (doublet), t (triplet), q (quartet), m (multiplet), and b (broad). Scalar coupling constants J are reported in Hertz (Hz). ^1^H and ^13^C NMR spectra were referenced to solvent peaks as reported in literature.

#### 4.2.3 Rheometry

Frequency sweep and strain sweep experiments were performed on a TA Instruments Discovery Hybrid Rheometer HR‐2. A parallel‐plate geometry with radius of 8 mm was used and coupled with a bottom plate, with a typical gap ranging between 1.5–1.7 mm to maintain a starting axial force of 0.05 N. Frequency sweep experiments were performed from 0.1 to 100 rad/s at 1% strain, which was first confirmed to be in the linear viscoelastic regime using strain sweep experiments. To avoid sample slippage on the bottom plate, sandpaper was applied using double‐sided Scotch tape. Gap height was corrected for sandpaper and tape thickness.

#### 4.2.4 Fourier Transform Infrared Spectroscopy (FTIR–ATR)

FTIR measurements were obtained on a Bruker Alpha II FTIR spectrometer with a Diamond Crystal attenuated total reflectance accessory at the MIT Department of Chemistry Instrumentation Facility.

#### 4.2.5 High Resolution Mass Spectrometry (HRMS)

HRMS measurements were obtained on a JEOL AccuTOF 4G LC‐plus equipped with an ionSense DART at the MIT Department of Chemistry Instrumentation Facility.

#### 4.2.6 Nitrogen Adsorption Isotherm/BET

Brunauer–Emmett–Teller (BET) surface areas of polymer powders were measured using N_2_ sorption at 77 K using a Micromeritics ASAP 2020 analyzer. Powder samples were degassed under high vacuum at 120°C for at least 4 h prior to analysis.

#### 4.2.7 Small Angle X‐Ray Scattering/Wide Angle X‐Ray Scattering (SAXS/WAXS)

SAXS and WAXS measurements for C2‐low were conducted at the Argonne National Laboratory Advanced Photon Source (APS) at beamline 12‐ID‐B. Samples of the material were loaded into hermetically sealed Al wells between two Kapton films under Ar atmosphere for measurement. All data were acquired with an x‐ray energy of 13.3 keV and an exposure time of 0.5 s. SAXS and WAXS patterns were acquired simultaneously on a Pilatus 2 M and Pilatus 300K detector respectively. The sample to detector distance for SAXS was 2 m. An in‐situ heating plate was used for temperature control, and samples were allowed to equilibrate at each temperature for 5 min before data acquisition. 2D data were radially integrated to yield 1D traces, and the x‐ray scattering peak positions were extracted using a Wolfram Mathematica script. Additional SAXS measurements were performed as a function of temperature on FAS‐PIM‐1Li polymer at MIT with a SAXSLAB system using a Rigaku 002 microfocus Cu source (*λ* = 1.54 Å), and a Pilatus 300K detector. Multiple sample‐detector distances were used, covering a total q range of 0.015–2.4 Å^−1^. Samples were prepared in the Argon glove box in air‐tight cells from Xenocs (part number SC‐ST‐1‐HSFX) with mica windows, and heated using a Linkam HFSX350‐GI stage. The films were measured at each temperature for 20 min. The X‐ray scattering peak positions were determined by fitting the scattering peaks to pseudo‐Voigt functions.

#### 4.2.8 Differential Scanning Calorimetry (DSC)

Pieces (5–15 mg) of heat‐pressed and dried polymer/SN films were loaded into TA Tzero Hermetic Aluminum DSC pans. DSC experiments were conducted on a TA Instruments Discovery 250 under a constant flow of nitrogen. An initial heating cycle from room temperature to 140°C at 10°C/min was run to remove thermal history followed by a subsequent cycle of cooling and heating at 10°C/min and cooling at 5°C/min between 140°C and −40°C were conducted. Peaks and glass transition temperatures were extracted using TA Instruments TRIOS software.

#### 4.2.9 Thermogravimetric Analysis (TGA)

Pieces (1–10 mg) of dried polymer/SN films were loaded into tared platinum pans. TGA was conducted on a TA Instruments Discovery 5500 from room temperature to 500°C at 10°C/min under a nitrogen environment and analyzed using TA instruments TRIOS software.

#### 4.2.10 Electrochemical Impedance Spectroscopy (EIS)

Polymer/SN blends were hot pressed at 65°C under 2 tons of pressure between stainless steel electrodes (1.55 cm in diameter), and dried under high vacuum at room temperature for 24 h. Samples transferred to an argon filled glovebox without exposure to atmosphere and placed in an air‐tight conductivity cell showed no distinguishable difference from conductivity cells constructed under air. Ionic conductivity was determined using electrochemical impedance spectroscopy, which was measured using a VMP3 (BioLogic) potentiostat, with a frequency range of 1 Hz–1 MHz with 50 mV sinusoidal amplitude. The cell was measured upon heating and cooling, between 30°C and 80°C, with 10°C steps. A thermostat chamber (SU‐241, Espec) was used for heating the cell, and the cell was equilibrated at each temperature for 1 h before conductivity was measured. After completion of conductivity measurements, cells were opened, and final thickness measurements were made of the sample pellet via calipers. The conductivity was calculated $\sigma =\frac{t}{R_{b}A}$ where R is the resistance determined from fitting the impedance results to an equivalent circuit model (Figure S23), t is the thickness, and *A* is the cross‐sectional area.

#### 4.2.11 Fabrication of Polysulfonimide/SN Blends

Succinonitrile was added to polysulfonimide powder in the desired 5 molar equivalents per repeat unit in a scintillation vial. A stir bar and anhydrous acetone (1 mL/50 mg polymer) were added and the mixture was stirred overnight to ensure homogenous mixing. Acetone was removed under reduced pressure at 32 ‐°C by rotary evaporator, and then the solid was further dried at room temperature under high vacuum for two days to yield a stiff glassy composite.

#### 4.2.12 Li^+^ Transference Number

To estimate the transference number, the Bruce–Vincent Approach was used [60]. This method can be applied in this work as: i) the **C1** electrolyte is solid/immobilized, that is, no convection is present to disrupt the establishment of a linear concentration gradient; and (ii) the applied DC voltage in the measurement is small (<10 mV, so that the potential distribution across the cell could be linearized) [80].

The equation used to calculate transference number is as follows:

$$t_{Li^{+}}=\frac{i_{\mathrm{SS}}}{i_{0}}\;\frac{\Delta V-i_{0}R_{\mathrm{CT}}^{0}}{\Delta V-i_{\mathrm{SS}}R_{\mathrm{CT}}^{\mathrm{SS}}}$$

where Δ*V* is the applied DC voltage, $R_{\mathrm{CT}}^{0}$ and $R_{\mathrm{CT}}^{\mathrm{SS}}$ are the interphasial resistances measured at the initial and the steady state, respectively. i_SS_ is the current at the steady state and i_0_ the initial current which can be more accurately calculated from AC impedance instead of being derived from DC polarization curve. Detailed experimental conditions, data analysis steps, and sample calculations can be found in the Supporting Information.

## Author Contributions

The manuscript was written through contributions of all authors. All authors have given approval to the final version of the manuscript.

## Conflicts of Interest

The authors declare no conflicts of interest.

## Supporting information



Materials and instruments; details of methods; synthesis and characterization of molecules and polymers; additional experimental results, details and discussions.
**Supporting File**: anie74944‐sup‐0001‐SuppMat.docx.

## Data Availability

The data that support the findings of this study are available from the corresponding author upon reasonable request.
