# Supplementary material for: Synthesis of Ladder and Flexible Polysulfonimides Directly From Anionic Monomers: Investigating Structure–Property Convergence in Succinonitrile–Polymer Electrolyte Blends
Source: Angew Chem Int Ed Engl. 2026 Sep 26;65(46):e8257644. doi: 10.1002/anie.8257644 (PMC13749335; doi:10.1002/anie.8257644)
Supplement: Supplementary file 1 [file ANIE-65-e8257644-s001.docx]

## *Supporting information for:*

**Synthesis of Ladder and Flexible Polysulfonimides Directly from Anionic Monomers: Investigating Structure–Property Convergence in Succinonitrile–Polymer Electrolyte Blends**

Abraham Herzog-Arbeitman,^1^ Pablo A. Leon,^2^ Benedikt S. Schreib,^1^ KyuJung Jun,^2^ Jurgis Ruza,^2^ Vanesa Munoz Perales, ^1,2^ Yang Shao-Horn,^1,2^ Rafael Gomez-Bombarelli,^2^ Jeremiah A. Johnson^1^*

**Affiliations**

^1^Department of Chemistry, Massachusetts Institute of Technology, 77 Massachusetts Avenue, Cambridge, MA 02139, U.S.A.

^2^Department of Materials Science and Engineering, Massachusetts Institute of Technology, 77 Massachusetts Avenue, Cambridge, MA 02139, U.S.A.

*Correspondence to: [jaj2109@mit.edu](mailto:jaj2109@mit.edu)

Materials and Methods

Materials

General solvents (dichloromethane, dimethyl formamide, hexane, ethyl acetate, acetone, toluene, propylene carbonate) were purchased from Sigma Aldrich. 4-Trifluoromethyl, 2,3,5,6-tetrafluorobenzene thiol was purchased from Ambeed Inc., and used as received. Trifluoromethanesulfonamide was purchased from Fischer Sci. and used as received. Succinonitrile was purchased from Fischer Sci and sublimed twice before use. All other reagents were purchased from Sigma Aldrich and used without further purification unless otherwise noted. All deuterated solvents were purchased from Cambridge Isotope Laboratories. FAST-C was synthesized using literature procedure.^1^ 4-Trifluoromethyl, 2,3,5,6-tetrafluorobenzene sulfonyl bromide was synthesized using literature procedure.^2^ Tetra(trimethylsilyl) 5,5',6,6'-tetrahydroxy-3,3,3',3'-tetramethyl-1,1'-spirobisindane (TTSBI-TMS) was synthesized according to literature procedure,^3^ and distilled prior to use.

Methods

*Column chromatography.* Flash silica column chromatography was performed using Biotage Isolera One with Accelerated Chromatographic IsolationTM flash chromatography system using KP-Sil SNAP cartridges at recommended flow rates.

*Solution-state nuclear magnetic resonance (NMR) spectroscopy*. 1H and 13C spectra were collected at rt (25 °C) and recorded on either a two-channel Bruker Avance-III HD Nanobay 400 MHz spectrometer, three-channel Bruker Avance Neo 500 MHz spectrometer, or four-channel Bruker Avance Neo 600 MHz spectrometer. Data were analyzed with MestReNova Version: 14.1.0-24037. Chemical shifts are expressed in parts per million (ppm), and splitting patterns are designated as s (singlet), d (doublet), t (triplet), q (quartet), m (multiplet), and b (broad). Scalar coupling constants J are reported in Hertz (Hz). ^1^H and ^13^C NMR spectra were referenced to solvent peaks as reported in literature.

*Rheometry*. Frequency sweep and strain sweep experiments were performed on a TA Instruments Discovery Hybrid Rheometer HR-2. A parallel-plate geometry with radius of 8 mm was used and coupled with a bottom plate, with a typical gap ranging between 1.5–1.7 mm to maintain a starting axial force of 0.05 N. Frequency sweep experiments were performed from 0.1 to 100 rad/s at 1% strain, which was first confirmed to be in the linear viscoelastic regime using strain sweep experiments. Experiments were performed at 25 °C, with negligible solvent evaporation during the typical measurement time (< 15 min).

*Fourier Transform Infrared Spectroscopy (FTIR-ATR):* FTIR measurements were obtained on a Bruker Alpha II FTIR spectrometer with a Diamond Crystal attenuated total reflectance accessory at the MIT Department of Chemistry Instrumentation Facility.

*High Resolution Mass Spectrometry (HRMS):* HRMS measurements were obtained on a JEOL AccuTOF 4G LC-plus equipped with an ionSense DART at the MIT Department of Chemistry Instrumentation Facility.

*Nitrogen Adsorption Isotherm/BET.* Brunauer-Emmett-Teller (BET) surface areas of polymer powders were measured using N_2_ sorption at 77 K using a Micromeritics ASAP 2020 analyzer. Powder samples were degassed under high vacuum at 120 °C for at least 4 hours prior to analysis.

*Small Angle X-ray Scattering/Wide Angle X-ray Scattering (SAXS/WAXS).* SAXS and WAXS measurements for all samples but neat FAS-PIM-1 were conducted at the Argonne National Laboratory Advanced Photon Source (APS) at beamline 12-ID-B. Samples of the material were loaded into hermetically sealed Al wells between two Kapton films under Ar atmosphere for measurement. All data were acquired with an X-ray energy of 13.3 keV and an exposure time of 0.5 seconds. SAXS and WAXS patterns were acquired simultaneously on a Pilatus 2M and Pilatus 300K detector respectively. The sample to detector distance for SAXS was 2 meters. An in-situ heating plate was used for temperature control, and samples were allowed to equilibrate at each temperature for 5 minutes before data acquisition. 2D data were radially integrated to yield 1D traces, and the X-ray scattering peak positions were extracted using a Wolfram Mathematica script. Additional SAXS measurements were performed as a function of temperature on FAS-PIM-1Li polymer at MIT with a SAXSLAB system using a Rigaku 002 microfocus Cu source (λ=1.54 Å), and a Pilatus 300K detector. Multiple sample-detector distances were used, covering a total q range of 0.015–2.4 Å^-1^. Samples were prepared in the Argon glove box in air-tight cells from Xenocs (part number SC-ST-1-HSFX) with mica windows and heated using a Linkam HFSX350-GI stage. The films were measured at each temperature for 20 minutes. All samples were prepared as Li salts. The X-ray scattering peak positions were determined by fitting the scattering peaks to pseudo-Voigt functions.

*Differential Scanning Calorimetry (DSC).* Pieces (~5–15 mg) of heat-pressed and dried polymer/SN films were loaded into TA Tzero Hermetic Aluminum DSC pans. DSC experiments were conducted on a TA Instruments Discovery 250 under a constant flow of nitrogen. An initial heating cycle from room temperature to 140 °C at 10 °C/min was run to remove thermal history followed by a subsequent cycle of cooling and heating at 10 °C/min and cooling at 5 °C/min between 140 °C and -40 °C were conducted. Peaks and glass transition temperatures were extracted using TA Instruments TRIOS software.

*Thermogravimetric Analysis (TGA).* Pieces (~1–10 mg) of dried polymer/SN films were loaded into tared platinum pans. TGA was conducted on a TA Instruments Discovery 5500 from room temperature to 500 °C at 10 °C/min under a nitrogen environment and analyzed using TA instruments TRIOS software.

*Electrochemical Impedance Spectroscopy (EIS).* Polymer/SN blends were hot pressed at 65°C under 2 tons of pressure between stainless steel electrodes (1.55 cm in diameter), and dried under high vacuum at room temperature for 24 hours. Samples transferred to an argon filled glovebox without exposure to atmosphere, and placed in an air-tight conductivity cell showed no distinguishable difference from conductivity cells constucted under air. Ionic conductivity was determined using electrochemical impedance spectroscopy, which was measured using a VMP3 (Bio-Logic) potentiostat, with a frequency range of 1 Hz to 1 MHz with 50 mV sinusoidal amplitude. The cell was measured upon heating and cooling, between 30 –80 °C, with 10°C steps. A thermostat chamber (SU-241, Espec) was used for heating the cell, and the cell was equilibrated at each temperature for 1 h before conductivity was measured. After completion of conductivity measurements, cells were opened, and final thickness measurements were made of the sample pellet via calipers. The conductivity was calculated $\sigma=\frac{t}{R_{b}A}$ where t is the thickness, and A is the cross-sectional area. R is the resistance determined from fitting the impedance results to an equivalent circuit model shown below (R = resistor; Q = constant phase element):

*Fabrication of polysulfonimide/SN blends.* Succinonitrile was added to polysulfonimide powder in the desired 5 molar equivalents for repeat unit in a scintillation vial. A stir bar and anhydrous acetone (1 mL/50 mg polymer) were added and the mixture was stirred overnight to ensure homogenous mixing. Acetone was removed under reduced pressure at 32°C by rotary evaporator, and then the solid was further dried at room temperature under high vacuum for two days to yield a stiff glassy composite.

*Li^+^ Transference number.* To estimate the transference number, the Bruce-Vincent Approach was used^4^. This method can be applied in this work as: i) the FAS-PIM-1/SN electrolyte is solid/immobilized, i.e., no convection is present to disrupt the establishment of a linear concentration gradient; and (ii) the applied DC voltage in the measurement is small (<10 mV, so that the potential distribution across the cell could be linearized).^5^ The procedure consists of the following steps:

1. Lithium-metal (Li^0^) foil electrodes were passivated by submerging in N,N-dimethylfluorosulfonamide for 3 days at room temperature in an Ar-filled glovebox.
2. A symmetric electrochemical cell was assembled consisting of the polymer electrolyte placed in between a pair of passivated lithium-metal (Li^0^) foil electrodes in an Ar-filled glovebox, and sealed in a coin cell.
3. The electrochemical cell was placed inside a climatic chamber, and equilibrated to 60 °C.
4. Open circuit voltage was applied to assess stability before starting the experiment.
5. In the case of lithium symmetric cells, one must also consider the presence of interphases and the evolution of interfacial resistance with time. Thus, electrochemical impedance spectroscopy is also performed before and after the polarization step, to obtain the interfacial transfer resistance, which is later included in the calculation. EIS before polarization is also used to calculate initial current with higher accuracy than the first measured point after polarization, as proposed by Evans et al.^4^
6. A 10 mV direct current (DC) voltage was applied to the cell to initiate polarization, and the resulting current is measured over time until it stabilizes at a constant value, (here, 1.44 $\mu A$ after smoothing, *vide infra*), referred to as the "steady state." Initially, the measured current i_0_ arises from the movement of both cations and anions. However, once steady state is reached, the current i_SS_ is attributed solely to cation transport, as the electrode polarization inhibits anion migration.

Chronoamperometry data smoothing:

As can be seen from Fig. S17A, typical Cottrell current decay behavior during polarization is partially masked by an oscillation with frequency ~ 0.4 mHz, which we attribute to the temperature control system within our laboratory. To obtain more accurate steady state current information, chronoamperometry data was smoothed using a 2400 s moving average (just larger than the oscillation period). $i_{SS}$ was simply determined using a trailing average. Initial current was calculated as follows:

$$i_{0}^{EIS}=\frac{\Delta V}{{R^{0}}_{b}+{R^{0}}_{CT}}=\frac{0.010 V}{35222.9 Ohm+28110.2 Ohm}=0.158 \mu A$$

The equation used to calculate transference number is as follows:

$$t_{Li^{+}}=\frac{i_{\mathrm{SS}}}{i_{0}} \frac{\Delta V-i_{0}R_{\mathrm{CT}}^{0}}{\Delta V-i_{\mathrm{SS}}R_{\mathrm{CT}}^{\mathrm{SS}}}$$

where ΔV is the applied DC voltage, $R_{\mathrm{CT}}^{0}$ and $R_{\mathrm{CT}}^{\mathrm{SS}}$ are the interphasial resistances measured at the initial and the steady state, respectively.

$$t_{Li^{+}}=\frac{i_{\mathrm{SS}}}{i_{0}} \frac{\Delta V-i_{0}R_{\mathrm{CT}}^{0}}{\Delta V-i_{\mathrm{SS}}R_{\mathrm{CT}}^{\mathrm{SS}}}=\frac{144 nA}{158 nA}*\frac{0.010 V-158 nA*28110 Ohm}{0.010 V-144 nA*27652 Ohm}=0.85$$

We note that the visible background oscillation also contributes to error in measurements of ${R^{0}}_{CT}$, ${R^{SS}}_{CT}$, and $i_{0}$ (through ${R^{0}}_{bulk}$ and ${R^{0}}_{CT}$). While approximate corrections to these values could be made, for example by scaling resistances using the ratio of smoothed current to raw instantaneous current at the beginning and end of the chronoamperometry strep (a <1.5% difference), these changes increase measured t_Li+_ only ~0.01, well within error (calculations not shown). Error propagation analysis indicates that the measurement error of 0.1 originates in the potentiostat current resolution limit, therefore additional corrections will not provide a more accurate estimate.

| Source | Value | Uncertainty | $\partial t_{li^{+}}/\partial x$ | Contribution to $\sigma_{tLi+}$^†^ | Rel. effect | % of variance |
| --- | --- | --- | --- | --- | --- | --- |
| ${R^{0}}_{b}$ | 35,222.9 Ω | 714.7 Ω | 2.404×10⁻⁵ /Ω | 0.0172 | 1 | 2.9 |
| ${R^{0}}_{CT}$ | 28,110.2 Ω | 689.5 Ω | 0 | 0 | 0 | 0 |
| ${R^{SS}}_{CT}$ | 27,652.6 Ω | 866.3 Ω | 2.035×10⁻⁵ /Ω | 0.0176 | .665 | 3 |
| $i_{\mathrm{SS}}$ | 144.4 nA | 10 nA* | 9.761×10⁶ /A | 0.0981 | 1.665 | 94.1 |

*instrument resolution at applied 10 uA range. Statistical error of moving average ~0.1 nA, disregarded.

^†^ Error contributions must be combined in quadrature, yielding overall error of 0.1.

*Density Functional Theory (DFT) interaction energy calculations.*

Lithium-anion interaction energies were computed through a scheme of quantum chemical geometry optimizations. Initial geometries of each anion were generated using RDKit^6^**** to convert SMILES strings into Cartesian coordinates. For each anion, a lithium cation was randomly placed between 1.5 and 4 Å of the anionic center. These clusters were refined using semi-empirical tight-binding DFT (GFN2- xTB),3^7^ using the ORCA simulation package. Then, the conformer-rotamer sampling tool (CREST)^8^ is used through ORCA with the same tight-binding potential to further sample poses. Final energies of these conformers were computed as the final step of a geometry optimization with the ωB97X-D3 range-separated hybrid functional^9^ with the def2-SVP basis set^10^ in vacuum using the qchem simulation package.^11^ The XC_SMART_GRID method with default parameters was used to determine the grid at each SCF cycle during optimization. Separately, the minimum energy conformation of each anion (i.e., without a cation) was determined using the same sequence. The final interaction energy was computed as E(interaction) = E(cluster) − (E(Li+ ) − E(anion)), where E(x) is the energy of the minimum energy configuration.

*FAS-PIM-1Li/SN and FAS-P-2 molecular dynamics simulations.* The OPLS-AA (Optimized Potentials for Liquid Simulations All Atom) force field is used for the molecular dynamics simulations^12,13^. The OPLS-AA forcefield is widely used for simulations of solvation and kinetic properties for both known and novel polymer electrolyte systems^14,15^. All succinonitrile parameters and the covalent interactions for the polymers are parametrized through the LigParGen server for OPLS-AA, while point charges were determined through RESP charges.^16–18^ To obtain the partial charges with the RESP fitting scheme, an ensemble of 50 conformations was generated for a 3–6 repeat unit *n*-mer with a short vacuum MD trajectory using the OPLS-AA interatomic potential parameterized with LigParGen under the *NVT* ensemble. We performed single point calculations for point charge determination with the ORCA^19^** quantum chemistry package on these conformations with the ωB97X-D range-separated hybrid functional with the def2-TZVP basis set and D3 dispersion correction.^20^ To obtain the RESP charges from the single point calculations, we used the Multiwfn electronic wave function analysis software.^21^

Ion charges were scaled by 0.9 to account for the overrepresentation of Coulomb interactions known for non-polarizable classical force fields. All simulations were performed with the GROMACS simulation package^22^ using a cutoff of 12 Å for explicit nonbonded interactions. Coulomb interactions beyond this are calculated with the Particle Mesh Ewald method. Full equilibration details can be seen below. Constant temperature and volume simulations were conducted using a timestep of 2 fs and a Nose-Hoover thermostat. Hydrogen bonds were constrained using the LINCS algorithm.

Table S1: Equilibration simulation workflow.

| Step | Time [ns] | Cumulative Time [ns] | Timestep [fs] | Temperature  [K] | Pressure  [bar] |
| --- | --- | --- | --- | --- | --- |
|  | Relaxation |  |  |  |  |
| 0 | 1 | (1) | 1 | 10 | 10 |
| 0.5 | 2 | (3) | 1 | 300 | 100 |
| 1 | 2 | 2 | 1 | 500 | 1 |
| 2 | 2 | 4 | 1 | 500 | 100 |
| 3 | 2 | 6 | 2 | 450 | 100 |
| 4 | 2 | 8 | 2 | 450 | 1 |
| 5 | 2 | 10 | 2 | 353 | 1 |
| 6 | 2 | 12 | 2 | 450 | 1 |
| 7 | 2 | 14 | 2 | 353 | 1 |
| 8 | 2 | 16 | 2 | 400 | 1 |
| 9 | 2 | 18 | 2 | 353 | 1 |
| 10 | 2 | 20 | 2 | 450 | 1 |
| 11 | 10 | 30 | 2 | 353 | 1 |
| Production | 100 | NVT | 2 | 353 | - |

As the FAS-PIM-1 polymer backbone is very rigid, a significant equilibration procedure was performed to ensure proper sampling. ChemDraw was used to create an initial 2D conformation of a single polymer chain of length 28 repeat units. This structure was parameterized with the OPLS-AA forcefield using LigParGen. Then, a sequence of constrained geometry optimizations was performed using OpenMM to create a 3D conformation of the single chain. First, bonds and angles were relaxed without nonbonded interactions. Then, the structure was relaxed with all interactions. We used Packmol to create an initial simulation containing 15 polymer chains, enough lithium to create charge neutrality (420 Li ions), and 5 times the number of SN as Li to match experiments for a total of 49380 and 28380 atoms with and without SN, respectively. Three separate initial configurations were created for each system.

We then ran a series of pressure and annealing cycles inspired by Molinari et al^23^ to condense the simulation to experimental densities using the GROMACS simulation package. Holding times and temperatures were increased in order to account for the stiffer backbones of the FAS polymers compared to the original polyethylene oxide backbones of the original method. We determined a charge scaling of 0.9 to be the best balance of densities and conductivity predictions as seen in Fig. S27 to account for the overrepresentation of Coulomb interactions seen for nonpolarizable classical force fields^3,4,5^. After equilibration, 100 nanoseconds of constant temperature and volume simulations were conducted for each system, with diffusivity and conductivity computed and averaged for each lithium in the systems with moving window averages up to half the length of the simulation due to decreasing statistics for larger windows.

Diffusivities of Li^+^, SN, and sulfonimide groups were determined by first tracking the sliding window-averaged mean squared displacement (MSD) of individual Li^+^ ions, N from SN, and O or N- from the sulfonimide groups, respectively, over time windows $dt$. The diffusivity was then computed by fitting the slope of the MSD vs. time graph in the linear diffusive regime. To extract contributions to total Li^+^ diffusion from different mechanisms (e.g. SN exchange, anion handoff, reptation, or hopping to/from the pore edge), MSD increments over $dt$ were categorized and separated based on changes in the solvation sphere of each Li ion which intuitively define each mechanism using a strategy similar to the analysis used for PEO-LiTFSI by Leon et al.^23^

A convex-hull based algorithm is used at each timestep to determine the oxygen and nitrogen in the first solvation shell of each lithium as explained in Leon et al. The SN and sulfonimide groups within the first solvation shell at the beginning and end of the observed time interval are compared to ascribe a physically meaningful lithium transport mechanism to each displacement. If only SN are in the first solvation shell, the lithium is labelled as experiencing a fully SN-solvated state. On the other hand, if any of the solvation shell is ascribed to a sulfonimide oxygen or nitrogen, the lithium is considered to be on the pore edge. When a lithium is at the pore edge at the beginning and end of the observed time interval, the transition is further refined depending on whether a new sulfonimide group enters or leaves the solvation shell.

In order to accurately capture the contributions of each mechanism, the duration of the time window dt must be sufficient to capture diffusion events of each mechanism but not be overly long such that multiple diffusion events of different mechanistic origins are attributed to a single process. Analysis discussed herein utilize $dt=100 ps$, which accurately distinguishes pore-center SN exchange processes. Results of mechanistic analysis at varying *dt*s from 10–10,000 ps is shown in Fig. S30 for completeness. Hops to and from the pore center were grouped with transport within the pore center for simplicity. The SN exchange and hopping to/from the pore edge mechanisms can no longer be resolved at timescales longer than 1000 ps for FAS-PIM-1 as seen by the collapsing of their respective per-event normalized effective diffusivity onto the per-event effective diffusivity of all events involving a purely SN-solvated state.

**Supplementary Figures S1 – S34**

**Fig. S1.** DFT calculated interaction energies for small-molecule analogues of FASP repeat units. Energies are calculated in vacuum.

**Fig. S2.** SEC Traces for FAS**-**PIM-1 and FAS-P-2 in DMF (0.025M LiBr).

**Fig. S3.** ^19^F NMR (470 MHz, DMSO) of polysulfonimides synthesized, and their fluorinated starting materials.

**Fig. S4.** Verification of cation exchange to Li from crude mixture. A) Elemental composition of FAS-PIM-1 as assessed by X-ray photoelectron spectroscopy. B) ^7^Li (194 MHz) and ^23^Na (132 MHz) NMR in DMSO-d_6_ of FAS-PIM-1 and FAS-P-2.

**Fig. S5.** Thermal analysis of polysulfonimides and composites. Top: TGA. Bottom: DSC. DSC curves depict the cooling cycle after an initial 10°C/s heat ramp to 140°C. Curves are offset for readability.

**Fig. S6.** Structures of FAS-PIM-1 repeat segment (top) and FAS-P-2 repeat unit (bottom) showing relevant length scales potentially responsible for observed peaks in SAXS/WAXS

**Fig. S7.** EIS experiments between 30–80 °C on pure FAS-PIM-1Li films, showing low conductivity.

**Fig. S8. ^1^H NMR characterization of C1 after drying.** Full spectrum in DMSO-d6. Inset: zoomed view including 1.8–2.7 ppm, showing no visible acetone signal at 2.09 ppm. Aromatic regions are integrated to 4 H, corresponding to 1 repeat unit.

**Fig. S9.** SAXS/WAXS of FASPs and C1 and C2.

**Fig. S10.** Simulated pore distributions and simulation snapshots for C1 and C2, with unfilled FAS-P-2 for comparison. Below: snapshots of C1 and C2. Polymer backbone shown in gray; accessible volume for SN shown in green.

**Fig. S11**. Density values for PIM simulations with SN as a function of ion charge scaling. Conductivity was measured using both the Nernst-Einstein formalism as well as with the cluster Nernst-Einstein formalism as described by France-Lanord^10^ to account for correlated ion motions.

**Fig. S12.** Temperature and amplitude sweeps of C1 (left) and C2 (right). (A, B) Tan delta temperature sweeps at 10 Hz. (C, D) Storage and loss moduli temperature sweeps at 10 Hz. Average of independent triplicates are shown in dark lines, individual samples shown with thin lines. (E and F) Strain amplitude sweeps at 10 Hz, 25 °C. Amplitude sweeps were conducted prior to annealing, such that any disruption to sample internal structure at high strain was thermally erased prior to frequency sweeps, heating ramps, etc.

**Fig. S13**. Rheology frequency sweeps (0.5% strain) for C1 (left) and C2 (right). A) 25 °C, before and after a 2°C/min ramp to 120°C with rapid cooling. Solid lines represent sweeps before heat ramp, dotted lines indicate sweeps after heating. B) 120 °C. Different colors indicate different sample replicates.

**Fig. S14.** Flammability test of C1 film. A) before exposure to flame; B) film put directly into open flame; C) Flame is removed. Slight char and smoking was observed in (B) but no ignition occurred.

**Fig. S15**. Reprocessing of the composite and reclamation of components. A) schematic of reprocessing and physico-chemical recycling of components. B) SEC (0.025 M LiBr in DMF) of original FAS-PIM-1 and FAS-PIM-1 recycled from C1. C) ^19^F NMR spectra of original and recycled FAS-PIM-1.

| **Component** | **MW** | **Eq** | **mmol** | **mass (mg)** |
| --- | --- | --- | --- | --- |
| SN | 80 | 0.185 | 1.295 | 103.6 |
| LiTFSI | 287.09 | 0.037 | 0.259 | 74.35631 |
| PEO | 44 | 1 | 7 | 308 |

**Fig. S16**. Rheology of PEO/SN/LiTFSI composite. A) Temperature sweep at 10 Hz. B) Frequency sweep at 25 °C. C) Frequency sweep at 120°C. Below: formulation of composite.

| **[Before]** | Fit Value | Std. Error | **[After]** | Fit Value | Std. Error |
| --- | --- | --- | --- | --- | --- |
| R_bulk_ | 35222.9 /Ohm | 714.7 /Ohm | R_bulk_ | 35319.9 /Ohm | 878.1 /Ohm |
| R_CT_ | 28110.2 /Ohm | 689.5 /Ohm | R_CT_ | 27652.6 /Ohm | 866.3 /Ohm |
| CPE1 | 0.316 /nF | 0.0183 /nF | CPE1 | 0.304 /nF | 0.019 /nF |
| $\alpha$1 | 0.897 | 0.0042 | $\alpha$1 | 0.899 | 0.0050 |
| CPE2 | 173 /nF | 12.8 /nF | CPE2 | 173 /nF | 14.3 /nF |
| $\alpha$2 | 0.589 | 0.0047 | $\alpha$2 | 0.586 | 0.0077 |

**Fig. S17**. Transference number measurement for C1 using the Bruce-Vincent method at 60 ºC. Top: EIS before and after 10mV DC bias applied for chronoamperometry, and chronoamperometry applied to a Li/C1/Li symmetric coin cell (electrode diameter 12.7 mm, C1 electrolyte thickness 0.2 mm). Black depicts the raw current response; red depicts a smoothed curve (2400 s moving average) used to extract the steady-state current i_SS_ = 0.145 µA. **EIS was u**sed to determine the interfacial charge-transfer resistance R_CT = R_CT1 + R_CT2 entering the Bruce-Vincent equation. Markers show experimental data; lines show fits to the equivalent circuit model, with fit values below. n = 1 replicate.

**Fig. S18**. Ashby plot for comparing reported modulus and conductivity properties of organic Li-ion single-ion conductors in literature. Triangles indicate materials containing small molecule solvents or dopants (with molar mass below 500 Da); black borders indicate crosslinked materials or materials otherwise not reprocessible. All listed materials reported t_Li+_ > 0.85. G′ data are reported when available but are substituted with E′/3 when not. While this substitution is necessarily an approximation, it appreciably increases the pool of data available, and we anticipate deviations of E′/3 from G′ to be within an order of magnitude, generally much less as Poisson ratios for many polymer electrolytes are between 0.3–0.5.^24–26^ Data were adapted from the following references: (1),^27^ (2),^28^ (3),^29^ (4),^30^ (5),^30^ (6),^31^ (7),^32^ (8),^33^ (9),^34^ (10),^35^ (11),^36^ (12),^37^ (13),^38^ (14),^39^ (15),^40^ (16),^41^ (17),^42^ (18).^43^ This figure is not intended to be a comprehensive report of literature materials, but rather a representative picture of reported property combinations at the time of writing.

**Fig. S19.** Full conductivity replicates for C1, C2, and C2-low.

**Fig. S20.** Rheology temperature sweep of C2-low at 10 Hz.

**Fig. S21.** FTIR spectroscopy characterization of SN-cation interaction. Spectra were normalized to the intensity of the free nitrile stretch (diagram inset) at 2252 cm^-1^.

**Fig. S22.** Comparison of simulation of FAS-PIM-1 with and without SN. (A and B) distribution of distances between Li and polymer backbone sulfonimides A) with SN and B) without. (C and D) radial distribution functions for Li+ C) without SN and D) with SN. E) Mean squared displacement of all Li^+^ and polymer backbones without SN present. Diffusivity is shown to be effectively negligible. Here, “with SN” corresponds to 5 molar equivalents, 36.5 wt. %.

**Fig. S23**. Distance distribution between Li^+^ and possible coordination partners. Top: C1. Bottom: C2.

**Fig. S24.** Sample timecourse of a pore-edge bound Li^+^ in MD simulation of C1. Top: distance to sulfonimide oxygen atoms during the timecourse. Distance to two sulfonimides are tracked, and the sulfonimide-sulfonimide distance is tracked (in gold) to illustrate Li handoff when two anions are in close proximity. Middle: Li^+^ displacement over time. Steps in the displacement coincide with sulfonimide movement. Bottom: complete Li^+^ coordination partners at each time is shown by a colored bar in a row corresponding to a particular labeled coordinating partner. If Li^+^ is coordinated by two groups on a single species (sulfonimide), that bar is pink.

**Fig. S25.** Sample timecourse of a pore-center Li^+^ in MD simulation of C1. Top: distance to specific SN ligands the timecourse. Distance to each SN is shown in a different color. The Li^+^ is shown to navigate many SN molecules during the timecourse. Multiple changes to the coordination shell occur when the color switches more rapidly. Middle: Li^+^ displacement over time. Steps in the displacement coincide with larger changes in the SN coordination environment (multiple rapid SN exchanges). Orange segments signify sulfonimide coordination (contact with pore edge); blue segments indicate pore center diffusion. Bottom: complete Li^+^ coordination partners at each time is shown by a colored bar in a row corresponding to a particular labeled coordinating partner. If Li^+^ is coordinated by two groups on a single species (sulfonimide), that bar is pink.

**Fig. S26**. Sample timecourse of a pore-center Li^+^ in MD simulation of C2. Top: distance to specific SN ligands the timecourse. Distance to each SN is shown in a different color. The Li^+^ is shown to navigate many SN molecules during the timecourse. Multiple changes to the coordination shell occur when the color switches more rapidly. Orange segments signify sulfonimide coordination (contact with pore edge); blue segments indicate pore center diffusion.

 **Fig. S27.** Deconstruction of mechanistic contributions to Li^+^ mean squared displacement (MSD) within simulations of C1 and C2. Mechanisms are distinguished through changes in Li+ solvation environments over a time window dt of 100 ps. “SN -> SN” indicates pore center diffusion; “Ntf -> Ntf change” indicates sulfonimide handoff; “SN <-> Ntf” indicates hops to or from the pore edge; “Ntf -> Ntf same” indicates movement of a Li-NTf ion pair; “Ntf -> Ntf” indicates the total of all pore-edge pathways; “SN involved” refers to the total of pore center and hops to or from the edge. Contributions are not normalized by the number of Li^+^ which undergo that type of diffusion to better show which environments contribute most to diffusion.

**Fig. S28.** Density and conductivity values for PIM simulations as a function of ion charge scaling. Conductivity was measured using both the Nernst-Einstein formalism as well as with the cluster Nernst-Einstein formalism as described by France-Lanord^10^ to account for correlated ion motions. Densities reproduced from Fig. S6.

**Fig. S29**. Impedance spectra of a passivated Li/C1/Li symmetrical cell over time. The interfacial and bulk components of the resistance can be seen to be stable, indicating SEI formation.

**Fig. S30**. Mechanistic decomposition of Li^+^ diffusion for C1 and C2 as defined by solvation changes over different time windows dt. (A and B) Frequency of occurrence of each diffusion event. (C and D) Total diffusivity of each mechanism. (E and F) Intrinsic diffusivity of each mechanism, i.e. diffusivity normalized by the frequency of occurrence of each event. As dt increases, frequent diffusion events such as SN exchanges become unresolvable as their effects on the final Li+ solvation environment are erased, leading to intrinsic diffusivity trending towards the global average, total diffusivity decreasing with frequency. For this work, dt = 100 ps was kept for all analyses.

Synthesis*:*

**FAST-B** (Na salt) was synthesized using a modified literature procedure. A 250 mL round-bottom flask with magnetic stir bar was charged with 24g (12.6 equiv, 226 mmol) Na_2_CO_3_, and flame-dried. 6.5g (1 equiv, 18 mmol) 4-trifluoromethyl, 2,3,5,6-tetrafluorobenzene sulfonyl bromide and 6.7g (2.5 equiv, 45 mmol) trifluoromethanesulfonamide were added. 85 mL of toluene and 40 mL of EtOAc were added, and the suspension was sonicated for 6 hrs, and then allowed to stir overnight at room temperature. Then the suspension was dried under decreased pressure and the residue was suspended in 80 mL EtOAc, and centrifuged to deposit solids. Supernatant was decanted, concentrated, and purified by column chromatography using a hexane/acetone gradient. Yield can be increased by dissolving deposited solids in minimal 1M aqueous HCl, extracting 5x with 20 mL EtOAc, and subsequent chromatographic purification. White powder product was recrystallized by dissolving in minimal acetone, adding 2 volumes of Et_2_O, and layering with hexanes to yield colorless crystals (92.5% yield). ^19^F NMR (376 MHz, acetone-d6, ppm, δ): -57.61, -79.58, -137.16, -141.80.

**FAS-PIM-1.** FAST-B Na 6.7g (1 equiv, 14.9 mmol), TTSBI-TMS 9.37g (1 equiv, 14.9 mmol), and oven-dried K_2_CO_3_ 6.2g (3 equiv, 44.5 mmol) were added to a 25 mL round-bottom flask and placed under inert atmosphere by 3 cycles of evacuation and backfilling with dry nitrogen. 8 mL anhydrous DMAc was added by syringe, and the mixture was briefly sonicated, then placed in a 40°C oil bath under active N_2_ flow with a vent needle for outlet, using a 18-gauge needle for inlet and outlet. N_2_ flow was kept low to avoid surface evaporation. After 1 hr, the solution was transferred to a 160°C oil bath for 4 hours, or until the solution was observed to thicken rapidly. The round-bottom flask was then removed from the bath, and the mixture was precipitated into saturated aqueous LiCl solution before filtering to yield a yellow solid. To ensure complete lithiation, the solid was refluxed in 50 mL saturated aqueous LiCl solution for two hours 3x, filtering and adding new solution each time, before refluxing with deionized water twice in the same way and finally drying under vacuum at 120°C overnight. Yield: 77%.

^19^F NMR (376 MHz, acetone-d6, ppm, δ): -54.46, -77.86. ^1^H NMR (500 MHz, DMSO) δ 6.92 (s, 1H), 6.85 (s, 2H), 6.34 (s, 3H), 2.29 (s, 2H), 2.11 (s, 3H), 1.34 (s, 7H), 1.27 (s, 10H).

**FAS-P-2**. FAST-C Na 2.8 g (1 equiv, 5.6 mmol), NDGA 1.69g (1 equiv, 5.6 mmol), and oven-dried K_2_CO_3_ 2.32 g (3 equiv, 16.8 mmol) were added to a 25 mL round-bottom flask and placed under inert atmosphere by 3 cycles of evacuation and backfilling with dry nitrogen. 8 mL anhydrous DMAc was added by syringe, and the mixture was briefly sonicated, then placed in a 40°C oil bath under active N_2_ flow with a vent needle for outlet, using a 18-gauge needle for inlet and outlet. N_2_ flow was kept low to avoid surface evaporation. After 1 hr, the solution was transferred to a 160°C oil bath for 4 hours, or until the solution was observed to thicken rapidly. The roundbottom was then removed from the bath, and the mixture was precipitated into saturated aqueous LiCl solution before filtering to yield a yellow solid. To ensure complete lithiation, the solid was refluxed in 50mL saturated aqueous LiCl solution for two hours 3x, filtering and adding new solution each time, before refluxing with deionized water twice in the same way and finally drying under vacuum at 120°C overnight. Yield: 52%.

^19^F NMR (376 MHz, DMSO) δ -137.05, -140.97, -155.71 (d, *J* = 283.2 Hz), -163.08, -163.66. ^1^H NMR (500 MHz, DMSO) δ 6.83 (s, 4H), 2.70 (s, 2H), 2.22 (s, 2H), 1.69 (s, 2H), 0.77 (s, 6H).

**P-3**. FAST-B Na 0.902 g (1 equiv, 2 mmol), NDGA (0.605 g, 1 equiv, 2 mmol), and oven-dried K_2_CO_3_ 0.829 g (3 equiv, 6 mmol) were added to a 25 mL round-bottom flask and placed under inert atmosphere by 3 cycles of evacuation and backfilling with dry nitrogen. 8 mL anhydrous DMAc was added by syringe, and the mixture was briefly sonicated, then placed in a 40°C oil bath under active N_2_ flow with a vent needle for outlet, using a 18-gauge needle for inlet and outlet. N_2_ flow was kept low to avoid surface evaporation. After 1 hr, the solution was transferred to a 160°C oil bath for 4 hours, or until the solution was observed to thicken rapidly. The round-bottom was then removed from the bath, and the mixture was precipitated into saturated aqueous LiCl solution before filtering to yield a yellow solid. To ensure complete lithiation, the solid was refluxed in 50 mL saturated aqueous LiCl solution for two hours 3x, filtering and adding new solution each time, before refluxing with deionized water twice in the same way and finally drying under vacuum at 120°C overnight. Yield: 31%.

^19^F NMR (376 MHz, DMSO) δ in ppm: -54.41 (t, *J* = 10.6 Hz), -77.84 (t, *J* = 13.7 Hz). ^1^H NMR (400 MHz, DMSO) δ 6.94 (s, 2H), 6.91 – 6.84 (m, 4H), 2.27 (s, 2H), 1.72 (s, 2H), 0.78 (s, 6H).

NMR Spectra*:*

**Fig. S31.** ^1^H NMR (500 MHz, DMSO-d_6_) spectrum of FAS-PIM-1.

**Fig. S32.** ^19^F NMR (376 MHz, DMSO-d_6_) spectrum of FAS-PIM-1.

**Fig. S33.** ^7^Li NMR (194 MHz, DMSO-d_6_) spectrum of FAS-PIM-1.

**Fig S34**. ^1^H NMR (500 MHz, DMSO-d_6_) spectrum of FAS-P-2.

**Fig. S35.** ^19^F NMR (376 MHz, DMSO-d_6_) spectrum of FAS-P-2

**Fig. S36.** ^7^Li NMR (194 MHz, DMSO-d_6_) spectrum of FAS-P-2.

1.	Stolberg, M. A. *et al.* Lamellar Ionenes with Highly Dissociative, Anionic Channels Provide Lower Barriers for Cation Transport. *J. Am. Chem. Soc.* **145**, 16200–16209 (2023).

2.	Platonov, V. E., Bredikhin, R. A., Maksimov, A. M. & Kireenkov, V. V. A novel and efficient method for the synthesis of polyfluoroarenesulfonyl bromides from polyfluoroarenethiols. *J. Fluor. Chem.* **131**, 13–16 (2010).

3.	Kricheldorf, H. R., Fritsch, D., Vakhtangishvili, L. & Schwarz, G. Cyclic Ladder Polymers by Polycondensation of Silylated Tetrahydroxy-tetramethylspirobisindane with 1,4-Dicyanotetrafluorobenzene. *Macromol. Chem. Phys.* **206**, 2239–2247 (2005).

4.	Evans, J., Vincent, C. A. & Bruce, P. G. Electrochemical measurement of transference numbers in polymer electrolytes. *Polymer* **28**, 2324–2328 (1987).

5.	Navigating the minefield of battery literature | Communications Materials. https://www.nature.com/articles/s43246-022-00251-5.

6.	RDKit. https://www.rdkit.org/.

7.	Bannwarth, C., Ehlert, S. & Grimme, S. GFN2-xTB—An Accurate and Broadly Parametrized Self-Consistent Tight-Binding Quantum Chemical Method with Multipole Electrostatics and Density-Dependent Dispersion Contributions. *J. Chem. Theory Comput.* **15**, 1652–1671 (2019).

8.	Pracht, P. *et al.* CREST—A program for the exploration of low-energy molecular chemical space. *J. Chem. Phys.* **160**, 114110 (2024).

9.	Lin, Y.-S., Li, G.-D., Mao, S.-P. & Chai, J.-D. Long-Range Corrected Hybrid Density Functionals with Improved Dispersion Corrections. *J. Chem. Theory Comput.* **9**, 263–272 (2013).

10.	Weigend, F. & Ahlrichs, R. Balanced basis sets of split valence, triple zeta valence and quadruple zeta valence quality for H to Rn: Design and assessment of accuracy. *Phys. Chem. Chem. Phys.* **7**, 3297–3305 (2005).

11.	Shao, Y. *et al.* Advances in molecular quantum chemistry contained in the Q-Chem 4 program package. *Mol. Phys.* **113**, 184–215 (2015).

12.	Jorgensen, W. L. & Tirado-Rives, J. The OPLS [optimized potentials for liquid simulations] potential functions for proteins, energy minimizations for crystals of cyclic peptides and crambin. *J. Am. Chem. Soc.* **110**, 1657–1666 (1988).

13.	Jorgensen, W. L. & Tirado-Rives, J. Potential energy functions for atomic-level simulations of water and organic and biomolecular systems. *Proc. Natl. Acad. Sci.* **102**, 6665–6670 (2005).

14.	Brooks, D. J., Merinov, B. V., Goddard, W. A. I., Kozinsky, B. & Mailoa, J. Atomistic Description of Ionic Diffusion in PEO–LiTFSI: Effect of Temperature, Molecular Weight, and Ionic Concentration. *Macromolecules* **51**, 8987–8995 (2018).

15.	Fang, C., Yu, X., Chakraborty, S., Balsara, N. P. & Wang, R. Molecular Origin of High Cation Transference in Mixtures of Poly(pentyl malonate) and Lithium Salt. *ACS Macro Lett.* 612–618 (2023) doi:10.1021/acsmacrolett.3c00041.

16.	Woods, R. J. & Chappelle, R. Restrained electrostatic potential atomic partial charges for condensed-phase simulations of carbohydrates. *J. Mol. Struct. THEOCHEM* **527**, 149–156 (2000).

17.	Dodda, L. S., Cabeza de Vaca, I., Tirado-Rives, J. & Jorgensen, W. L. LigParGen web server: an automatic OPLS-AA parameter generator for organic ligands. *Nucleic Acids Res.* **45**, W331–W336 (2017).

18.	Dodda, L. S., Vilseck, J. Z., Tirado-Rives, J. & Jorgensen, W. L. 1.14*CM1A-LBCC: Localized Bond-Charge Corrected CM1A Charges for Condensed-Phase Simulations. *J. Phys. Chem. B* **121**, 3864–3870 (2017).

19.	Neese, F. Software update: the ORCA program system, version 4.0. *WIREs Comput. Mol. Sci.* **8**, e1327 (2018).

20.	Neese, F. The ORCA program system. *WIREs Comput. Mol. Sci.* **2**, 73–78 (2012).

21.	Lu, T. & Chen, F. Multiwfn: A multifunctional wavefunction analyzer. *J. Comput. Chem.* **33**, 580–592 (2012).

22.	Van Der Spoel, D. *et al.* GROMACS: Fast, flexible, and free. *J. Comput. Chem.* **26**, 1701–1718 (2005).

23.	Leon, P., Jun, K., Gordiz, K., Shao-Horn, Y. & Gomez-Bombarelli, R. Mechanistic Decomposition of Ion Transport in Amorphous Polymer Electrolytes via Molecular Dynamics. Preprint at https://doi.org/10.26434/chemrxiv-2025-fs6gj (2025).

24.	Tavano, R. *et al.* Mechanical characterisation of a structural battery electrolyte. *Polymer* **312**, 127646 (2024).

25.	Monroe, C. & Newman, J. The Impact of Elastic Deformation on Deposition Kinetics at Lithium/Polymer Interfaces. *J. Electrochem. Soc.* **152**, A396 (2005).

26.	Gudla, H., Edström, K. & Zhang, C. Salt Effects on the Mechanical Properties of Ionic Conductive Polymer: A Molecular Dynamics Study. *ACS Mater. Au* **4**, 300–307 (2024).

27.	Kurian, M., Galvin, M. E., Trapa, P. E., Sadoway, D. R. & Mayes, A. M. Single-ion conducting polymer-silicate nanocomposite electrolytes for lithium battery applications. *Electrochimica Acta* **50**, 2125–2134 (2005).

28.	Filip, D., Asandulesa, M., Macocinschi, D., Aflori, M. & Vlad, S. Molecular dynamics, conductivity and morphology of sodium deoxycholate-based poly(ester ether)urethane ionomer biomaterials. *J. Mater. Sci.* **51**, 8516–8528 (2016).

29.	Wang, S.-W. & Colby, R. H. Linear Viscoelasticity and Cation Conduction in Polyurethane Sulfonate Ionomers with Ions in the Soft Segment–Multiphase Systems. *Macromolecules* **51**, 2767–2775 (2018).

30.	Jangu, C. *et al.* Sulfonimide-Containing Triblock Copolymers for Improved Conductivity and Mechanical Performance. *Macromolecules* **48**, 4520–4528 (2015).

31.	Li, S. *et al.* Single-Ion Homopolymer Electrolytes with High Transference Number Prepared by Click Chemistry and Photoinduced Metal-Free Atom-Transfer Radical Polymerization. *ACS Energy Lett.* **3**, 20–27 (2018).

32.	Lingua, G. *et al.* A New (Trifluoromethane)Sulfonylimide Single-Ion Conductor with PEG Spacer for All-Solid-State Lithium-Based Batteries. *ACS Mater. Lett.* **6**, 5429–5437 (2024).

33.	Shin, D.-M. *et al.* A Single-Ion Conducting Borate Network Polymer as a Viable Quasi-Solid Electrolyte for Lithium Metal Batteries. *Adv. Mater.* **32**, 1905771 (2020).

34.	Zhou, M. *et al.* Ultrathin Yet Robust Single Lithium-Ion Conducting Quasi-Solid-State Polymer-Brush Electrolytes Enable Ultralong-Life and Dendrite-Free Lithium-Metal Batteries. *Adv. Mater.* **33**, 2100943 (2021).

35.	Dreier, P. *et al.* Introduction of Trifluoromethanesulfonamide Groups in Poly(ethylene oxide): Ionic Conductivity of Single-Ion-Conducting Block Copolymer Electrolytes. *Macromolecules* **55**, 1342–1353 (2022).

36.	Villaluenga, I. *et al.* Compliant glass–polymer hybrid single ion-conducting electrolytes for lithium batteries. *Proc. Natl. Acad. Sci.* **113**, 52–57 (2016).

37.	Han, S. *et al.* Sequencing polymers to enable solid-state lithium batteries. *Nat. Mater.* **22**, 1515–1522 (2023).

38.	Weber, R. L. & Mahanthappa, M. K. Thiol–ene synthesis and characterization of lithium bis(malonato)borate single-ion conducting gel polymer electrolytes. *Soft Matter* **13**, 7633–7643 (2017).

39.	Porcarelli, L. *et al.* Single-Ion Conducting Polymer Nanoparticles as Functional Fillers for Solid Electrolytes in Lithium Metal Batteries. *ACS Appl. Mater. Interfaces* **13**, 54354–54362 (2021).

40.	Nikolakakou, G., Pantazidis, C., Sakellariou, G. & Glynos, E. Ion Conductivity–Shear Modulus Relationship of Single-Ion Solid Polymer Electrolytes Composed of Polyanionic Miktoarm Star Copolymers. *Macromolecules* **55**, 6131–6139 (2022).

41.	Nikolakakou, G. *et al.* Nanostructured Single-ion Polymer Blend Electrolytes Composed of Polyanionic Particles and Low Molecular Weight PEO. *ACS Macro Lett.* **12**, 1665–1671 (2023).

42.	Wang, H. *et al.* Single-Ion Polymer-in-Salt Electrolytes Enabling Percolating Ionic Nanoaggregates for Ambient-Temperature Solid-State Batteries. *J. Am. Chem. Soc.* **147**, 14554–14563 (2025).

43.	Yang, M. & Epps, T. H. I. Solid-State, Single-Ion Conducting, Polymer Blend Electrolytes with Enhanced Li+ Conduction, Electrochemical Stability, and Limiting Current Density. *Chem. Mater.* **36**, 1855–1869 (2024).
